# Adipocyte lipin 1 expression associates with human metabolic health and regulates systemic metabolism in mice

**DOI:** 10.1172/JCI169722

**Published:** 2024-10-15

**Authors:** Andrew LaPoint, Jason M. Singer, Daniel Ferguson, Trevor M. Shew, M. Katie Renkemeyer, Hector H. Palacios, Rachael L. Field, Sireesha Yerrathota, Roshan Kumari, Mahalakshmi Shankaran, Gordon I. Smith, Jun Yoshino, Mai He, Gary J. Patti, Marc K. Hellerstein, Samuel Klein, Jonathan R. Brestoff, E. Matthew Morris, Brian N. Finck, Andrew J. Lutkewitte

**Affiliations:** 1 Department of Medicine and; 2 Department of Pathology and Immunology, Washington University School of Medicine, St. Louis, Missouri, USA.; 3 Department of Medicine, Division of Endocrinology, Diabetes, and Clinical Pharmacology, University of Kansas Medical Center, Kansas City, Kansas, USA.; 4 Department of Nutritional Sciences and Toxicology, University of California, Berkeley, California, USA.; 5 Division of Nephrology, Department of Internal Medicine, Faculty of Medicine, Shimane University, Izumo, Shimane, Japan.; 6 Center for Integrated Kidney Research and Advance (IKRA), Shimane University, Izumo, Shimane, Japan.; 7 Department of Chemistry, Washington University School of Medicine, St. Louis, Missouri, USA.; 8 Department of Cell Biology and Physiology and; 9 KU Diabetes Institute and Kansas Center for Metabolism and Obesity, University of Kansas Medical Center, Kansas City, Kansas, USA.; 10 Center for Children’s Healthy Lifestyles and Nutrition, Kansas City, Missouri, USA.

**Keywords:** Hepatology, Metabolism, Diabetes, Insulin signaling, Obesity

## Abstract

Dysfunctional adipose tissue is believed to promote the development of hepatic steatosis and systemic insulin resistance, but many of the mechanisms involved are still unclear. Lipin 1 catalyzes the conversion of phosphatidic acid to diacylglycerol, the penultimate step of triglyceride synthesis, which is essential for lipid storage. Herein we found that adipose tissue *LPIN1* expression is decreased in people with obesity compared with lean subjects, and low *LPIN1* expression correlated with multi-tissue insulin resistance and increased rates of hepatic de novo lipogenesis. Comprehensive metabolic and multiomic phenotyping demonstrated that adipocyte-specific *Lpin1^–/–^* mice had a metabolically unhealthy phenotype, including liver and skeletal muscle insulin resistance, hepatic steatosis, increased hepatic de novo lipogenesis, and transcriptomic signatures of metabolically associated steatohepatitis that was exacerbated by high-fat diets. We conclude that adipocyte lipin 1–mediated lipid storage is vital for preserving adipose tissue and systemic metabolic health, and its loss predisposes mice to metabolically associated steatohepatitis.

## Abstract 2

Our data indicate that adipocyte Lpin1 deletion is sufficient to drive adipose tissue dysfunction leading to perturbations in systemic metabolism

## Graphical abstract

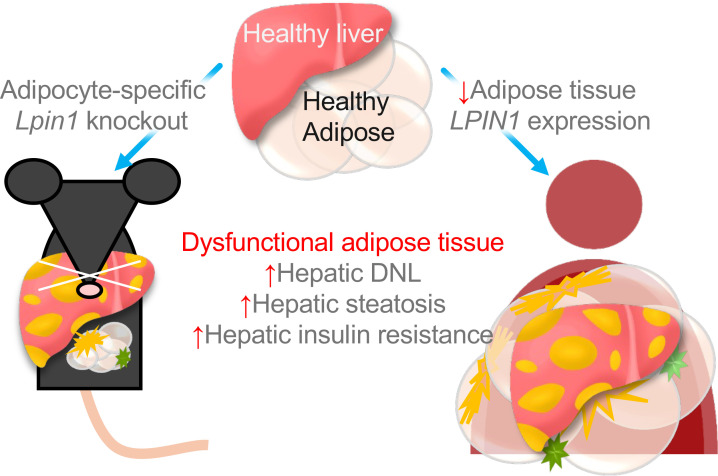

## Introduction

Adipose tissue plays essential roles in maintaining metabolic homeostasis by serving as a fat reservoir (lipogenesis) and a secretory organ that releases hormones and other biological factors that signal to distal organs. Although increased adipose tissue mass (obesity) is associated with an increased risk for hepatic steatosis, systemic insulin resistance, and cardiometabolic diseases, a subset of people with obesity do not develop these abnormalities and have metabolically healthy obesity (MHO), as opposed to metabolically unhealthy obesity (MUO) (1–3). These studies suggest that dysfunctional adipose tissue (defined here by adipose tissue insulin resistance, inflammation, and fibrosis), not adipose tissue mass, is linked to metabolic disease development. For example, whole-body insulin resistance is associated with decreased adipose tissue lipogenic gene expression in people with MUO compared with people with MHO (3–6). Alternatively, enhanced lipogenic capacity (i.e., lipid storage capacity) in adipose tissue prevents systemic insulin resistance in mouse models of obesity and is associated with increased insulin sensitivity in people (3, 7, 8). Furthermore, adipose tissue inflammation and fibrosis are also associated with insulin resistance and hepatic steatosis in people with obesity (9).

Adipocytes store lipids as triglycerides (TAGs) by the sequential acylation of glycerol-3-phosphate. The penultimate step of TAG synthesis, which is the conversion of phosphatidic acid to diacylglycerol (DAG), is carried out by phosphatidic acid phosphohydrolase (PAP) enzymes known as lipins (10, 11). There are 3 lipin family proteins (lipins 1, 2, and 3) that are differentially expressed in various tissues (12, 13). *Lpin1*, which encodes lipin 1, was first identified as the gene deleted in fatty liver dystrophic (*fld*) mice (12), which exhibit severe lipodystrophy associated with systemic metabolic abnormalities (14, 15). *Lpin1* is highly expressed in adipocytes and is essential for TAG synthesis in mice through its action as a PAP enzyme. Lipin 1 is also required to initiate adipogenic gene expression programs, likely via the effects of phosphatidic acid on signaling cascades and direct transcriptional regulation of gene expression in the nucleus (16–20). Conditional mice with adipocyte-specific expression of a truncated form of lipin 1, which lacked intrinsic PAP activity but retained activity as a transcriptional regulator, exhibited a lean phenotype (21, 22). These mice also exhibited insulin resistance despite being lean (22), whereas lipin 1–overexpressing mice had increased adiposity yet were insulin sensitive (23). Prior work has also identified correlations between high adipose tissue LPIN1 expression and insulin sensitivity in people (24–26). These studies suggest that high expression of lipin 1 in adipocytes may preserve adipose tissue function and protect from the development of fatty liver, insulin resistance, and other metabolic abnormalities.

Despite these striking effects of modulating of adipocyte lipin 1 activity on metabolic health, the mechanisms by which lipin 1 regulates systemic metabolism, specifically through adipose-liver crosstalk, are poorly understood. Moreover, prior studies of lipin 1 loss-of-function models were complicated by the expression of the truncated lipin 1 protein that retained activity as a transcriptional regulator (21). Herein, we used mice with a floxed *Lpin1* allele that was designed to completely delete the lipin 1 protein (27, 28), thereby generating mice with total loss of lipin 1 in adipocytes, and characterized their metabolic phenotype using rigorous metabolic phenotyping and multiomic approaches. In addition, we evaluated *LPIN1* gene expression in subcutaneous adipose tissue obtained from people who were metabolically healthy and lean (MHL) and people with MHO and MUO.

## Results

### Adipose tissue LPIN1 gene expression is decreased in people with MUO and correlates with insulin sensitivity and hepatic de novo lipogenesis.

**Figure 1:**
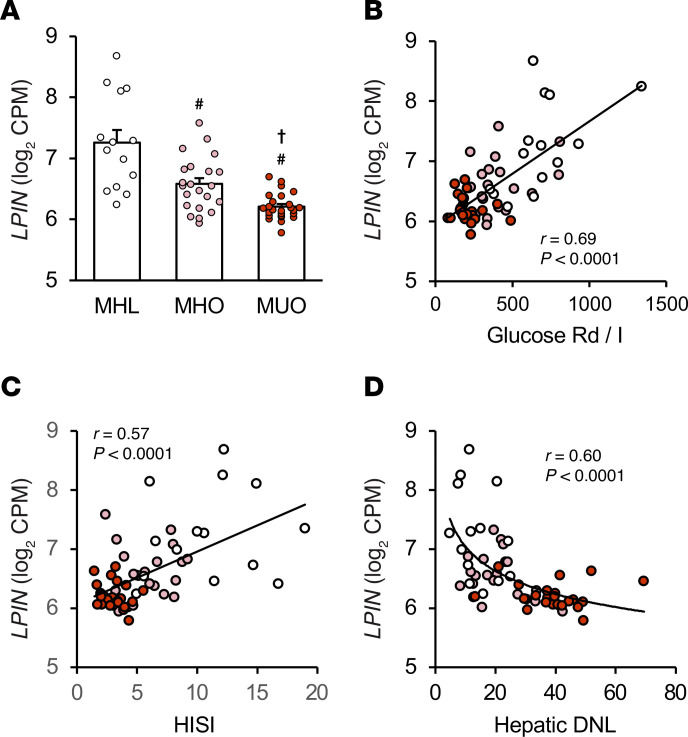
Abdominal adipose tissue *LPIN1* gene expression is decreased in people with metabolically unhealthy obesity and *LPIN1* expression correlates with metabolic health. (**A**) Gene expression of *LPIN1* from subcutaneous abdominal adipose tissue (SAAT) determined by RNA sequencing in metabolically healthy lean (MHL; *n* = 14), metabolically healthy obese (MHO; *n* = 22), and metabolically unhealthy obese (MUO; *n* = 25) groups. Data are expressed as means ± SEM. One-way ANOVA was used to compare *LPIN1* expression among MHL, MHO, and MUO groups with Fisher’s least significant difference post hoc procedure used to identify significant mean differences. ^#^*P* < 0.05 vs. MHL and †*P* < 0.05 vs. MUO. (**B**–**D**) Relationship between SAAT *LPIN1* expression and skeletal muscle insulin sensitivity (glucose rate of disappearance relative to plasma insulin concentration during the hyperinsulinemic-euglycemic clamp procedure [glucose Rd/I]), hepatic insulin sensitivity index (HISI), and contribution from hepatic de novo lipogenesis (DNL) to plasma triglyceride-palmitate. Logarithmic regression analysis was used to determine the line best fit to the data.

We compared *LPIN1* gene expression in subcutaneous abdominal adipose tissue from people who were metabolically healthy and lean (MHL), metabolically healthy with obesity (MHO), and metabolically unhealthy with obesity (MUO), stratified based on body mass index (BMI), glucose tolerance, hemoglobin A_1c_, and intrahepatic triglyceride (IHTG) content assessed by MRI (Supplemental Table 1; supplemental material available online with this article; https://doi.org/10.1172/JCI169722DS1) (11). Gene expression of *LPIN1* was highest in the MHL group and progressively decreased from MHL to the MHO and MUO groups (Figure 1A). In addition, *LPIN1* expression directly correlated with skeletal muscle insulin sensitivity, defined as the glucose disposal rate (Rd) per kilogram fat-free mass divided by plasma insulin during a hyperinsulinemic-euglycemic clamp procedure (1), and hepatic insulin sensitivity (assessed as the hepatic insulin sensitivity index), which is the reciprocal of the product of basal endogenous glucose production rate and basal plasma insulin concentration (Figure 1C) (2, 3). In contrast, *LPIN1* expression was inversely associated with rates of hepatic de novo lipogenesis (DNL) (Figure 1, B–D). These data demonstrate that adipose tissue *LPIN1* expression is associated with a healthy metabolic profile in people.

### Adipocyte-specific Lpin1-knockout mice have reduced fat mass.

To test the consequences of adipocyte *Lpin1* loss on systemic metabolism, we generated mice with adipocyte-specific *Lpin1* deletion (Adn-*Lpin1^–/–^*) by using *Lpin1*-floxed mice crossed with mice expressing adiponectin promoter–driven Cre recombinase. Prior work to delete *Lpin1* in adipocytes used a distinct line of floxed mice that resulted in expression of a truncated, partially functional protein (4, 5). However, in the present study we used mice with a floxed allele shown to induce the complete loss of lipin 1 protein in skeletal muscle and heart (6, 7). Loss of lipin 1 protein and *Lpin1* gene expression was restricted to adipose tissue (Supplemental Figure 1A). There was an increase in lipin 2 mRNA abundance and protein expression in adipose tissue of the Adn-*Lpin1^–/–^* mice (Supplemental Figure 1, B and C), though previous reports have indicated that the contribution of lipin 2 to adipose tissue PAP activity is minimal (8, 9). At 8 weeks of age, male and female Adn-*Lpin1^–/–^* mice had similar body weights and body composition on chow diet compared with littermate wild-type (WT) control mice (*Lpin1* floxed) (Supplemental Figure 2, A–D). Although female knockout mice were mildly hyperglycemic compared with WT controls, neither sex exhibited differences in an insulin tolerance test (ITT) (Supplemental Figure 2, E–H).

**Figure 2:**
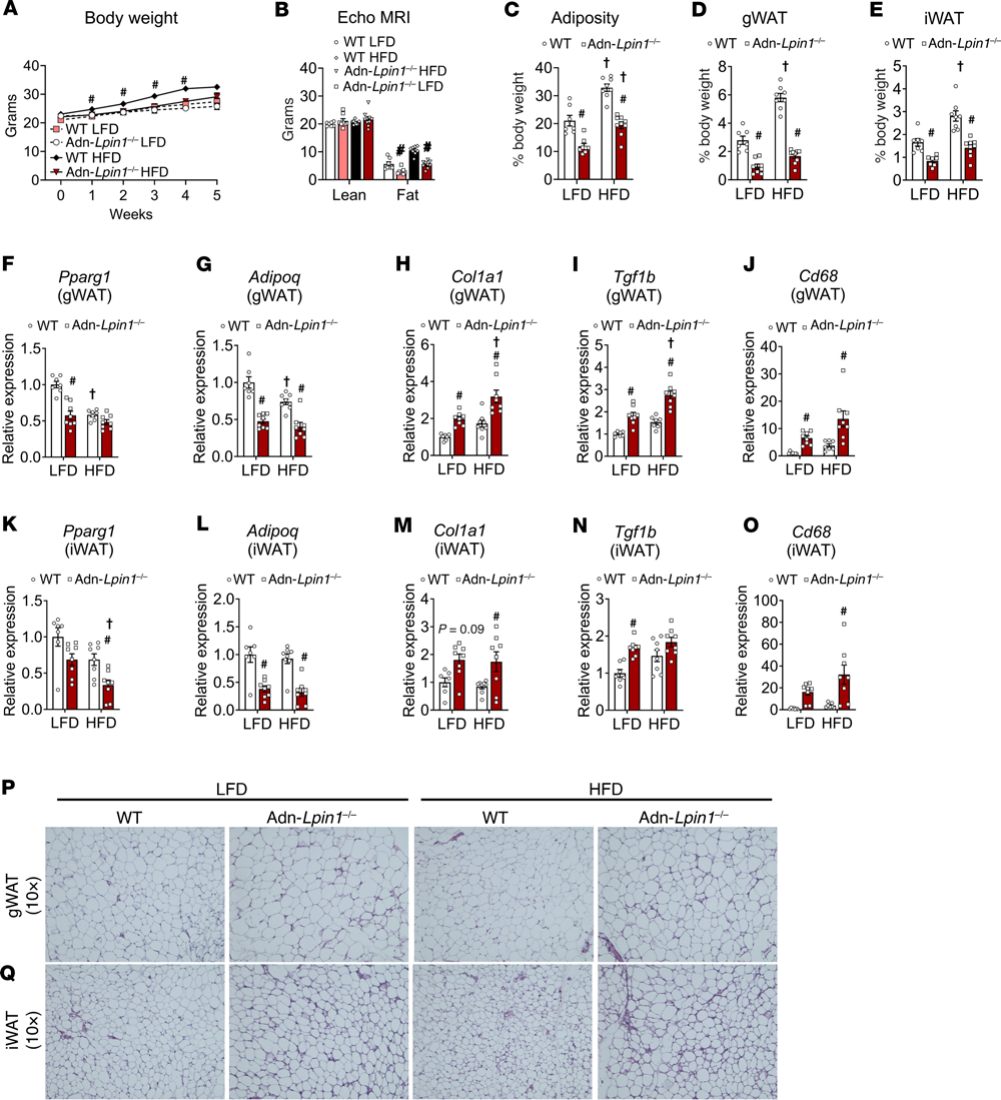
Adipocyte-specific *Lpin1-*knockout mice have reduced adiposity and signs of adipose tissue dysfunction. Eight-week-old male adipocyte-specific *Lpin1-*knockout mice (Adn-*Lpin1^–/–^*) and their wild-type littermate controls (WT) were fed either a 10% low-fat diet (LFD) or a 60% high-fat diet (HFD) for 5 weeks. Mice were fasted for 4 hours before sacrifice and tissue collection. (**A**) Body weights of mice during diet feeding. (**B**) Body composition was measured in fed mice after 5 weeks of diet via EchoMRI. (**C**) Adiposity was calculated as percentage fat mass/total body weight. (**D** and **E**) Tissue weight of gonadal white adipose tissue (gWAT) and inguinal white adipose tissue (iWAT) expressed as percentage body weight. (**F**–**O**) gWAT and iWAT gene expression was determined by quantitative PCR and is expressed as relative abundance. *Pparg1*, peroxisome proliferator–activated receptor γ-1; *Adipoq*, adiponectin; *Col1a1*, collagen type I α1 chain; *Tgfb1*, transforming growth factor-β1; *Cd68*, cluster of differentiation 68. (**P** and **Q**) Representative images at ×10 original magnification of gWAT and iWAT that were fixed in formalin before paraffin embedding, sectioning, and staining with H&E. Data are expressed as means ± SEM, and significance was determined by 2-way ANOVA with post hoc Tukey’s multiple-comparison tests. ^#^*P* < 0.05 for WT vs. Adn-*Lpin1^–/–^* and †*P* < 0.05 for LFD vs. HFD (*n* = 5–9).

Next, we fed male mice a high-fat diet (HFD; 60% kcal from fat) or a matched low-fat diet (LFD; 10% kcal from fat) to determine the effects of caloric excess on the phenotype. After 5 weeks of diet, WT mice fed HFD had a significant increase in body weight, gonadal and inguinal white adipose tissue (gWAT and iWAT) mass, and percentage adiposity compared with WT mice on LFD, while mice lacking adipocyte *Lpin1* gained less weight on the HFD and exhibited reduced fat pad weights compared with WT comparators on either diet (Figure 2, A–E). Adipose tissue from Adn-*Lpin1^–/–^* mice on both diets exhibited decreased expression of peroxisome proliferator–activated receptor γ-1 (*Pparg1*) and adiponectin (*Adipoq*) and increased expression of collagen type I α1 chain (*Col1a1*), transforming growth factor-β1 (*Tgfb1*), and cluster of differentiation 68 (*Cd68*; a macrophage marker) in comparison with WT control mice (Figure 2, F–O). Gross histology of gWAT and iWAT revealed increases in fibrosis and “crown-like” inflammatory structures in Adn-*Lpin1^–/–^* mice compared with WT mice, which was exacerbated by HFD feeding (Figure 2, P and Q).

Adipose tissue influences systemic metabolism through the regulated release of non-esterified fatty acids (NEFAs), glycerol, and various metabolic peptide hormones known as adipokines. In support of previous findings (4), mice lacking lipin 1 exhibited reduced plasma NEFA and glycerol concentrations, while TAG concentrations were unaffected by *Lpin1* loss (Supplemental Figure 3, A–C). Plasma adiponectin concentrations were significantly reduced in Adn-*Lpin1^–/–^* mice compared with WT mice on both diets (Supplemental Figure 3D). Plasma leptin and resistin concentrations tended to be lower in knockout mice but were not significantly reduced (Supplemental Figure 3F). Together these data suggest that loss of adipocyte lipin 1 is sufficient to cause adipose tissue dysfunction in mice.

### Adn-Lpin1^–/–^ mice have similar energetics to WT mice on either diet.

**Figure 3:**
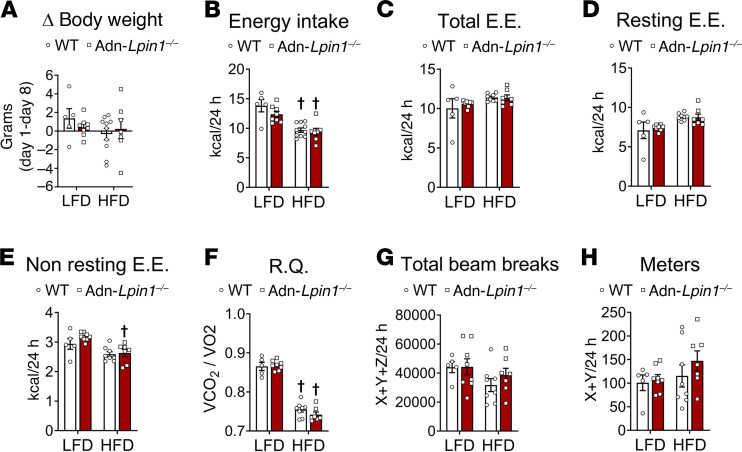
Adn-*Lpin1^–/–^* mice have similar metabolic energetics to WT mice. Eight-week old male mice were fed an LFD (*n* = 5–8) or HFD (*n* = 7–10) for 5 weeks before indirect calorimetry analysis. Mice were acclimated for 5 days before metabolic measurements. (**A**) Body weight changes over 8 days of indirect calorimetry housing. (**B**) Energy intake averaged over 8 days calculated as kcal/g/24 h. (**C**–**H**) All data were calculated as the 24-hour average over the last 3 days of indirect calorimetry housing. (**C**–**E**) Total energy expenditure (EE), resting EE, and non-resting EE were calculated as the 24-hour average for the last 3 days. (**F**) Respiratory quotient (RQ) (VCO_2_/VO_2_). (**G** and **H**) Activity was calculated as average beam breaks (*X*, *Y*, and *Z*). All data are expressed as means ± SEM, and significance was determined by 2-way ANOVA with post hoc Tukey’s multiple-comparisons tests. †*P* < 0.05 for LFD vs. HFD (*n* = 5–10).

Given that Adn-*Lpin1^–/–^* mice are leaner than their WT littermates, we used indirect calorimetry to assess energy intake, metabolic rates, substrate preferences, and activity. The mice were singly housed 2 weeks before housing in indirect calorimetry cages for 7–8 days. All the groups had similar non-significant changes in body weight during the entire 8-day period (Figure 3A). Five weeks of HFD feeding reduced energy intake in both genotypes (Figure 3B). The first 5 days of indirect calorimetry were considered an acclimation period, and metabolic data were collected during the final 48–72 hours of housing. Average 24-hour total energy expenditure and resting energy expenditure were similar between all groups (Figure 3, C and D), while non-resting energy expenditure was decreased by HFD feeding in Adn-*Lpin1^–/–^* (Figure 3E) The respiratory quotient was markedly reduced by the HFD in both genotypes (Figure 3F). Finally, there were no differences in cage activity between any groups (Figure 3, G and H).

### Adn-Lpin1^–/–^ mice are metabolically unhealthy.

Insulin and glucose tolerance tests were performed with WT and Adn-*Lpin1^–/–^* mice on either LFD or HFD. As observed with younger mice on a chow diet, 12- to 13-week-old Adn-*Lpin1^–/–^* mice fed an LFD exhibited similar plasma insulin and insulin (week 4 of diet) and glucose tolerance (week 5 of diet) compared with WT mice (Supplemental Figure 4). In contrast, Adn-*Lpin1^–/–^* mice fed an HFD had the highest fasting plasma insulin and blood glucose concentrations and were significantly insulin and glucose intolerant compared with all other mice after HFD feeding (Supplemental Figure 4). In contrast, this short-term HFD treatment did not induce glucose or insulin intolerance in HFD-fed WT mice compared with LFD-fed WT mice.

**Figure 4:**
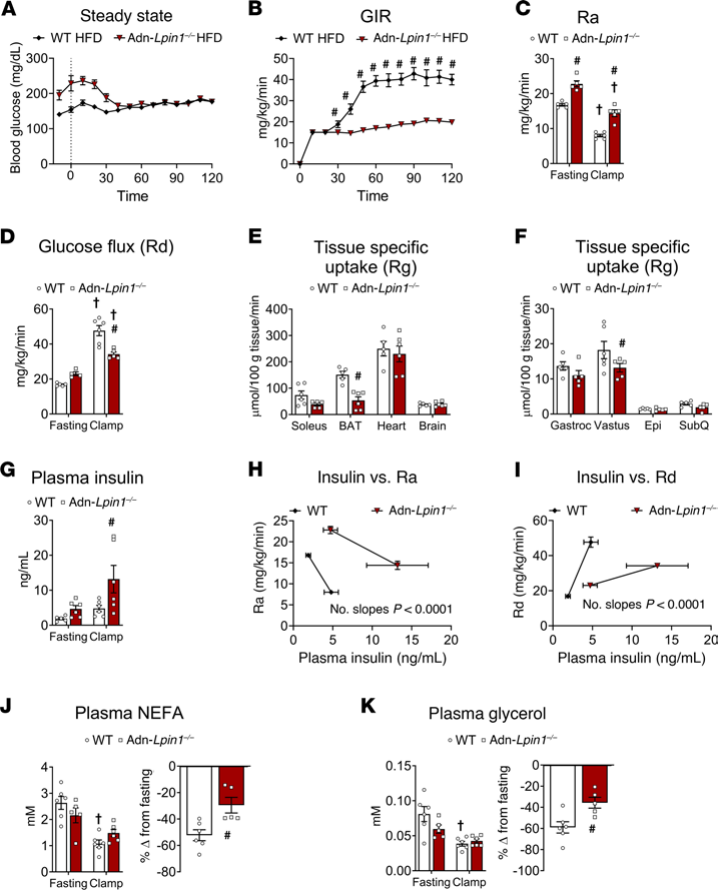
Adn-*Lpin1^–/–^* mice exhibit systemic insulin resistance on HFD. Eight-week-old male Adn-*Lpin1^–/–^* and WT control mice were fed a 60% HFD for 5 weeks. Five days before clamp, mice were catheterized and allowed to recover. Mice were fasted 5 hours before the clamp procedures as described in detail in Methods. (**A**) Blood glucose was monitored before and during the clamp and shows that both groups reached and sustained similar steady-state glucose concentrations during the clamp procedure. (**B**) Exogenous glucose infusion rates (GIR) were measured during the clamp procedure. (**C**) Endogenous glucose production (rate of appearance [Ra]) was determined from steady-state equations. (**D**) Whole-body glucose flux (disposal [Rd]) was determined from steady-state equations. (**E**) High tissue-specific glucose uptake (Rg). (**F**) Low tissue-specific glucose uptake (Rg). (**G**) Plasma insulin was measured by radioimmunoassay at –10 minutes for fasting, and 90 and 120 minutes were averaged for clamp values. (**H**) Ra was plotted against plasma insulin concentrations before and during the clamp. (**I**) Rd was plotted against plasma insulin concentrations before and during the clamp. (**J**) Plasma concentrations of non-esterified free fatty acids (NEFA) measured at fasting and clamp and represented as percent suppression from fasting. (**K**) Plasma concentrations of glycerol measured at fasting and during the clamp and represented as percent suppression from fasting. Data are expressed as means ± SEM, and significance was determined by Student’s *t* test (**A**, **B**, **E**, **F**, **H**, **I**, **J** inset, and **K** inset) or 2-way ANOVA with post hoc Tukey’s multiple-comparison tests where appropriate (**C**, **D**, **G**, **J**, and **K**). ^#^*P* < 0.05 for WT vs. Adn-*Lpin1^–/–^* and †*P* < 0.05 for LFD vs. HFD (*n* = 5–6).

WT and Adn-*Lpin1^–/–^* mice fed an HFD underwent hyperinsulinemic-euglycemic clamp studies, the gold standard to measure insulin sensitivity, to determine which tissues were insulin resistant (Figure 4). Adn-*Lpin1^–/–^* mice were hyperglycemic at the start of the clamp procedure and required significantly lower rates of exogenous glucose infusion to reach steady-state arterial blood glucose concentrations than WT mice (Figure 4, A and B). Fasting endogenous glucose production was elevated in Adn-*Lpin1^–/–^* mice, compared with WT mice, and insulin was less effective at suppressing glucose production during the clamp (Figure 4, C and D). Quantification of 2-deoxyglucose uptake into various tissues revealed that Adn-*Lpin1^–/–^* mice exhibited a substantial decrease in glucose disposal in subscapular brown adipose tissue (BAT) and vastus muscle, with a trend toward reduced uptake in soleus and gastrocnemius muscle (Figure 4, E and F). In clamped vastus muscle, the phosphorylation of insulin signaling proteins AKT and GSK3β was not different between genotypes, although insulin can increase glucose uptake in muscle independently of AKT activation (Supplemental Figure 4E) (10). Plasma insulin concentrations were also elevated during the clamp in Adn-*Lpin1^–/–^* mice (Figure 4G). Considering the rate of glucose appearance as a function of plasma insulin concentrations, displayed as the slope from fasted to clamp states, revealed a significant difference between genotypes, indicating that insulin was less effective at suppressing endogenous glucose production and providing further evidence of hepatic insulin resistance in Adn-*Lpin1^–/–^* mice (Figure 4H). Similarly, compared with WT mice, high insulin concentrations were less effective at increasing the glucose disposal rate in Adn-*Lpin1^–/–^* mice, indicating BAT and skeletal muscle insulin resistance (Figure 4I). Insulin was significantly more effective at suppressing plasma NEFA and glycerol concentrations in WT compared with Adn-*Lpin1^–/–^* mice, suggesting adipose tissue insulin resistance (Figure 4, J and K). These data suggest that loss of lipin 1 in adipocytes leads to systemic insulin resistance that impacts adipose, liver, and skeletal muscle.

### Loss of adipocyte Lpin1 leads to ectopic lipid accumulation and increased hepatic DNL.

**Figure 5:**
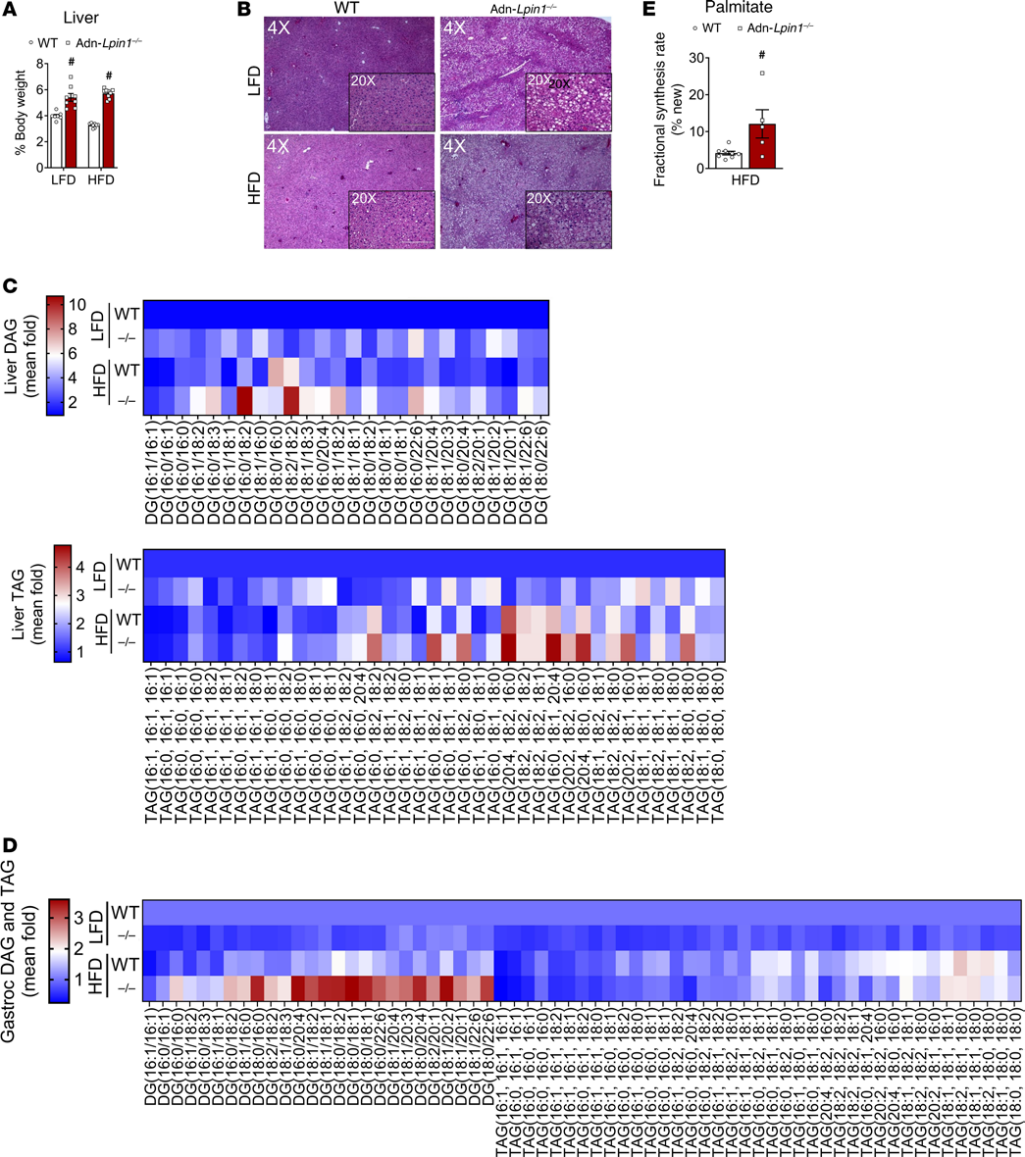
Loss of adipocyte *Lpin1* leads to severe hepatic lipid accumulation. Eight-week-old male Adn-*Lpin1^–/–^* and WT control mice were fed either a 10% LFD or a 60% HFD for 5 weeks. Mice were fasted for 4 hours before sacrifice and tissue collection. (**A**) Liver weight expressed as percentage of body weight. Data are expressed as means ± SEM, and significance was determined by 2-way ANOVA with post hoc Tukey’s multiple-comparison tests. ^#^*P* < 0.05 for WT vs. Adn-*Lpin1^–/–^* (*n* = 7–9). (**B**) Representative images at ×4 and ×20 original magnification of liver tissue that was fixed in formalin before paraffin embedding, sectioning, and staining with H&E. (**C** and **D**) Liver (**C**) and gastrocnemius (Gastroc) (**D**) diacylglycerol (DAG) and triglyceride (TAG) were extracted, and the relative abundance of each species was determined by liquid chromatography–tandem mass spectrometry (LC-MS/MS) against an internal standard. Data are expressed as mean fold change from the control LFD-fed mice (*n* = 7–9). (**E**) Total palmitate was analyzed by LC-MS for isotope mass abundance, and MIDA was used to calculate palmitate fractional synthesis rate (percent newly synthesized). Data are expressed as means ± SEM, and significance was determined by Student’s *t* test. ^#^*P* < 0.05 for WT vs. Adn-*Lpin1^–/–^* (*n* = 5–8).

On both LFD and HFD, Adn-*Lpin1^–/–^* mouse livers were enlarged compared with WT livers and appeared to accumulate lipids as indicated by the vacuolar appearance of sections after H&E staining (Figure 5, A and B). We performed targeted lipidomic analyses and found that in comparison with WT mice, intrahepatic DAG and TAG were much higher (10- and 5-fold, respectively) in Adn-*Lpin1^–/–^* mice fed LFD (Figure 5C). While HFD increased hepatic glycerolipid content in WT mice, the effect of HFD was more dramatic in Adn-*Lpin1^–/–^* mice than in WT mice (Figure 5C). Several TAG species with long polyunsaturated acyl chains were significantly increased, most notably 20:4 (Figure 5C). Similar increases in DAG, but not TAG, were observed in lipidomic analyses of gastrocnemius of these mice, indicating ectopic lipid accumulation in skeletal muscle as well (Figure 5D).

Adn-*Lpin1^–/–^* mice exhibited lower plasma lipids (Supplemental Figure 3, A–C) and only a trend toward lower TAG clearance following an oral olive oil gavage compared with littermates on chow (Supplemental Figure 2, I and K). These data suggest that the hepatic lipids may be derived from other sources in addition to adipocytes. Given that Adn-*Lpin1^–/–^* mice were insulin resistant, which is known to drive increased hepatic DNL (11), we sought to determine rates of DNL in Adn-*Lpin1^–/–^* mice on HFD. Indeed, isotopic tracing studies conducted using deuterated water confirmed an increase in the fractional synthesis rate of liver palmitate in Adn-*Lpin1^–/–^* mice compared with WT mice on HFD (Figure 5E). These data indicate that loss of *Lpin1* in adipocytes leads to hepatic steatosis in conjunction with increased rates of DNL.

### Bulk RNA sequencing analysis reveals significant changes in the expression of genes in pathways related to the transition from fatty liver to metabolically associated steatohepatitis.

**Figure 6:**
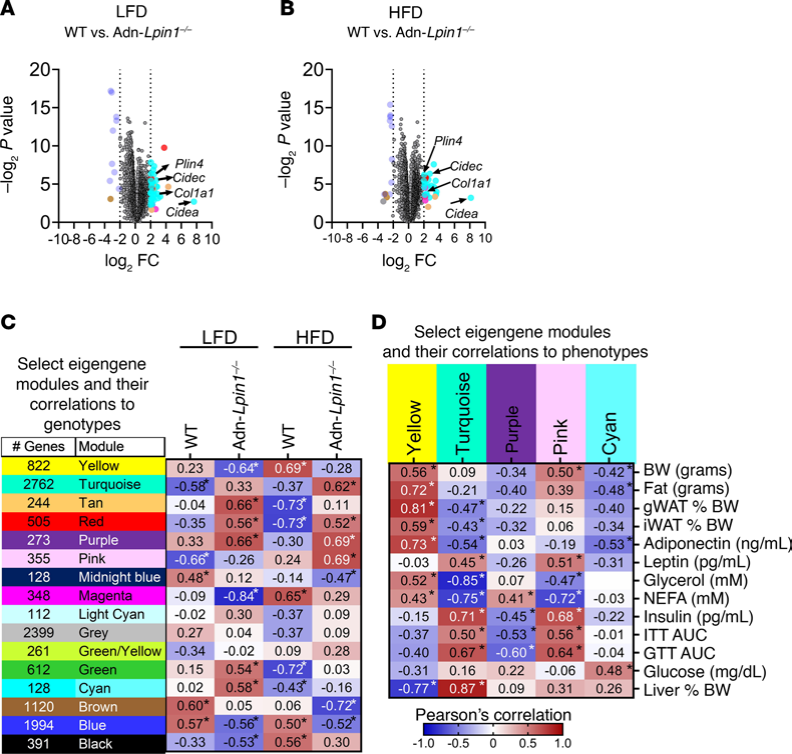
Bulk RNA sequencing analysis reveals significant changes in genetic pathways related to transition from early-stage fatty liver to MASH. Eight-week-old male Adn-*Lpin1^–/–^* and WT control mice were fed either a 10% LFD or a 60% HFD for 5 weeks. Mice were fasted for 4 hours before sacrifice, liver collection, RNA isolation, and bulk RNA sequencing. (**A** and **B**) Volcano plots of merged differential expression data were graphed as log_2_ fold change versus –log_10_ unadjusted *P* value. The color of the data points corresponds to the module eigengenes into which each gene clusters based on hierarchical clustering. (**C**) Select gene modules and the number of genes in each module from weighted gene coexpression network analysis (WGCNA). (**D**) Select signification modules and their correlation to phenotypic traits from the mice used to generate the WGCNA data. Pearson’s correlation coefficient was used to determine association with the WGCNA modules and trait data. *P < 0.05 (*n* = 6).

To identify the molecular pathways associated with insulin resistance and steatosis in Adn-*Lpin1^–/–^* mice, we performed bulk RNA sequencing in the liver (Figure 6 and Supplemental Figure 5). Principal component analyses demonstrated significant separation among the 4 groups with apparent gene changes represented by a heatmap (Supplemental Figure 5, A and B). There was a significant increase of genes associated with hepatic steatosis (*Cidea*, *Cidec*, and *Plin4*) and fibrosis (*Col1a1*) in Adn-*Lpin1^–/–^* livers on both diets compared with their diet-matched control mice (Figure 6, A and B). Next, we performed weighted gene coexpression network analysis (WGCNA) to identify highly correlated gene modules within the sequencing data (Figure 6C) (12). Several modules significantly correlated with the genotype of the mice and/or the diets (Figure 6C, select modules shown). The turquoise module contained the largest number of genes at 2,762, and most of the significantly upregulated genes in the volcano plots clustered to this module (Figure 6, A and B, and Supplemental Table 2). Additionally, the turquoise module was correlated positively with the HFD-fed Adn-*Lpin1^–/–^* mice and most negatively with the control LFD-fed mice (Figure 6C).

**Figure 7:**
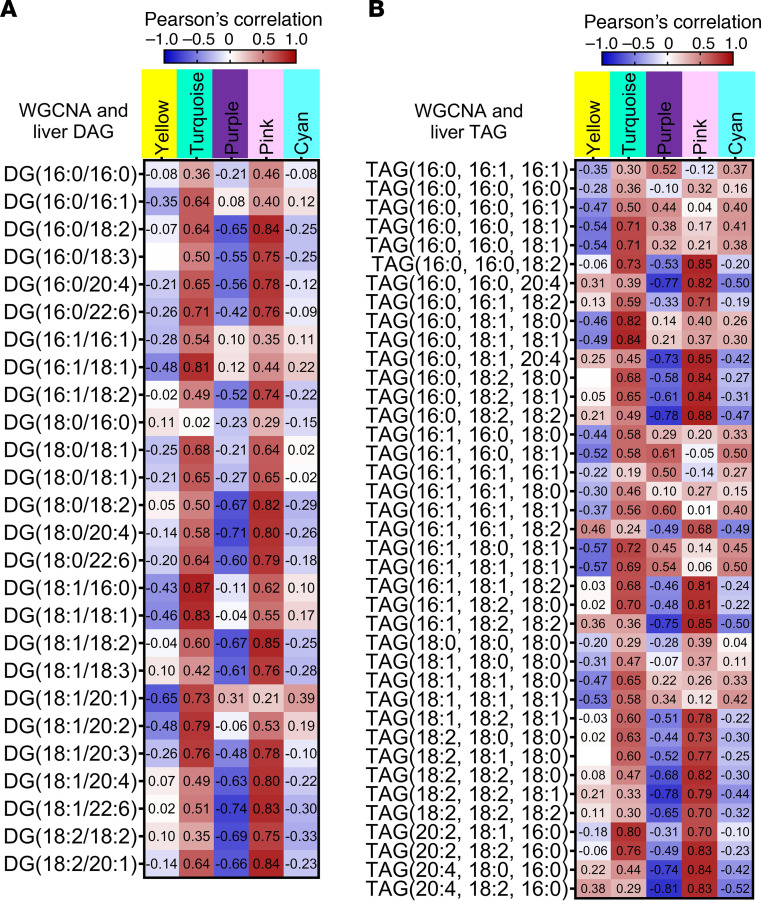
WGCNA analysis reveals significant correlations between modules and hepatic lipid species. Eight-week-old male Adn-*Lpin1^–/–^* and WT control mice were fed either a 10% LFD or a 60% HFD for 5 weeks. Mice were fasted for 4 hours before sacrifice, liver collection, RNA isolation, and bulk RNA sequencing. (**A** and **B**) Select signification modules and their correlation to DAG (**A**) and TAG (**B**) species from the lipidomics analysis. Pearson’s correlation coefficient was used to determine association with the WGCNA modules and trait data (*n* = 6).

We next combined WGCNA module sets with phenotypic traits from the mice used to generate the RNA sequencing data. Using Pearson’s correlation coefficient, we found several module sets that were significantly correlated to phenotypic data such as body composition, tissue size, plasma adiponectin, glycerol, and NEFAs (Figure 6D, select modules shown, and Supplemental Figure 6A). In addition, we compared our lipidomic data to identify gene modules that correlate with the increased DAG and TAG species seen in Adn-*Lpin1^–/–^* livers and discovered that many of these lipid species also had significant correlations to gene modules (Figure 7 and Supplemental Figure 6B). Specifically, the turquoise and pink modules positively correlated with plasma insulin levels, liver size, and most DAG and TAG species. Alternatively, these modules were negatively correlated to plasma glycerol and NEFAs (Figure 6D and Figure 7). The yellow module, which most negatively correlated to Adn-*Lpin1^–/–^* mice, and most DAG and TAG species had positive correlations for fat mass, adiponectin, plasma glycerol, and NEFA and negative correlations for ITT AUC, glucose tolerance test (GTT) AUC, blood glucose, and liver size (Figure 6, C and D, and Figure 7). The purple module was negatively associated with plasma insulin, ITT AUC, and GTT AUC and most long-chain polyunsaturated DAG and TAG species (Figure 6, C and D, and Figure 7).

Pathway analysis of the turquoise module genes revealed several significant Gene Ontology (GO) molecular pathways involved in extracellular matrix (ECM) components, lipid binding, and collagen binding (Supplemental Figure 7). In fact, one of the most significantly upregulated genes in Adn-*Lpin1^–/–^* livers is *Col1a1*, which clusters to the turquoise model and is a marker of activated stellate cells, the primary mediators of hepatic fibrosis (Figure 6, A and B, and Supplemental Table 2). Significant Kyoto Encyclopedia of Genes and Genomes (KEGG) pathways within the turquoise module included PI3K/AKT signaling, ECM-receptor interactions, and AGE/RAGE signaling pathway in diabetic complications (Supplemental Figure 7A). Pathway analysis of the pink module set included significant GO molecular function pathways involved in oxidoreductase activity, FAD/NAD binding, and significant GO biological pathways involved in metabolic and catabolic pathways specifically related to lipid metabolism (Supplemental Figure 7B). Yellow pathway analysis revealed significant changes in FoxO, mTOR, AMPK, and insulin signaling pathways, as well as regulation of kinase signaling and activity (Supplemental Figure 7C).

Targeting specific genes within each module, we found significant changes in genes with functions related to our observed phenotypes, including glucose metabolism, lipid metabolism, and insulin signaling (Supplemental Figure 8). De novo lipogenic genes (*Acly*, *Fasn*, *Acaca*, *Acacb*, and *Scd1-4*) clustered to the purple module and had the highest expression in the LFD-fed Adn-*Lpin1^–/–^* mice (Supplemental Figure 8). Although the purple module was negatively associated with many TAG and DAG species with longer, saturated acyl chains, most monounsaturated acyl chains were positively associated with the purple module (Figure 7 and Supplemental Figure 7E). Lastly, *Pck1* and *G6pc*, the rate-limiting enzymes of gluconeogenesis, were significantly upregulated in livers of the Adn-*Lpin1^–/–^* mice and clustered to the cyan module, which was positively associated with blood glucose levels (Supplemental Figure 7D and Supplemental Figure 8).

Because insulin resistance and liver disease progression are highly related (13), we confirmed the increased expression of several markers of metabolically associated steatohepatitis (MASH) development in Adn-*Lpin1^–/–^* mice by quantitative reverse transcriptase PCR. We demonstrated increased expression of genes related to fibrosis (*Col1a1*), matrix remodeling (*Timp1* and *Timp3*), and inflammation (*Spp1*, *Cd68*, *Il1b*, and *Tgfb1*) in comparison with WT mice on the same diet (Supplemental Figure 9A). Together these data suggest that the loss of adipocyte lipin 1 drives insulin resistance and increases early markers of hepatic fibrosis and MASH.

### Loss of adipocyte Lpin1 predisposes mice to liver injury and hepatic stellate cell activation.

**Figure 8:**
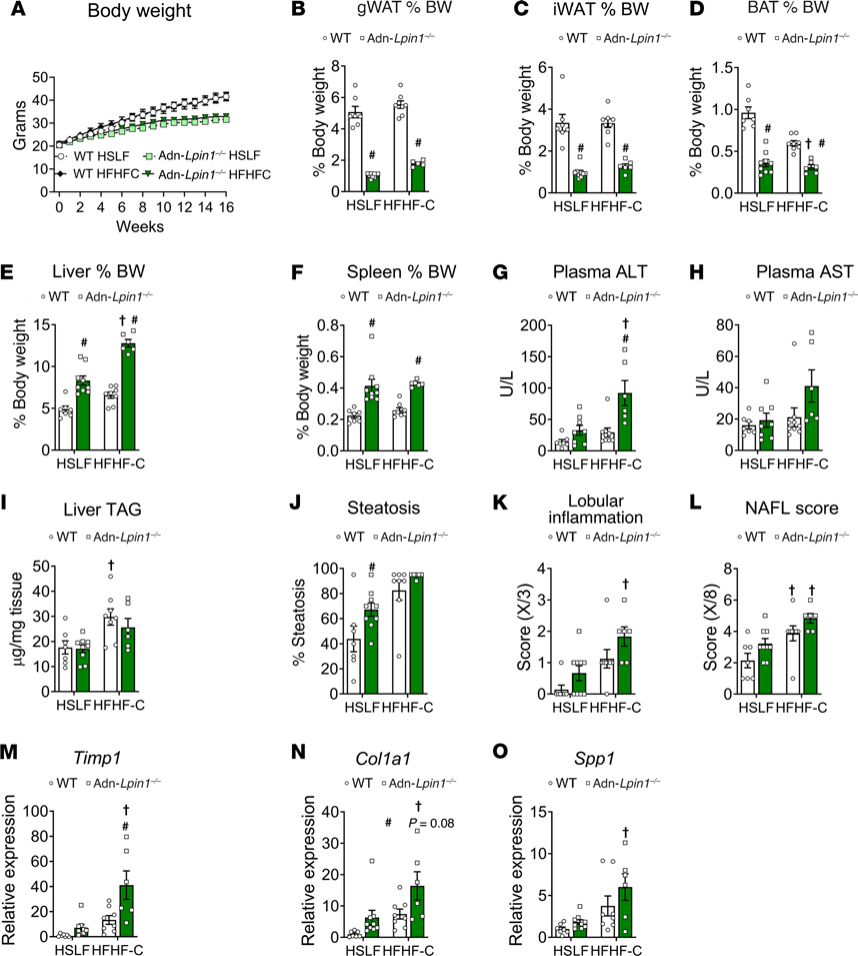
Loss of adipocyte *Lpin1* predisposes mice toward MASH. Eight-week-old male mice were fed a diet high in fructose (17 kcal %), fat (mostly palm oil, 40 kcal %), and cholesterol (2%) (HFHF-C) or a matched sucrose, high-sugar (dextrose), low-fat (10 kcal % fat) control diet (HSLF) for 16 weeks. Mice were fasted for 4 hours before sacrifice and tissue collection. (**A**) Weekly body weights. (**B**–**F**) Individual tissue weights expressed as percentage total body weight. (**G** and **H**) Plasma alanine transferase (ALT) and aspartate aminotransferase (AST) were measured using liquid kinetic assays. (**I**) Liver lipids were extracted and quantified using a colorimetric enzymatic assay. (**J**–**L**) H&E-stained liver sections were scored by an independent clinical pathologist. NAFL, nonalcoholic fatty liver. (**M**–**O**) Gene expression was determined by quantitative PCR and is expressed as relative abundance. *Timp1*, tissue inhibitor of metalloproteinase 1; *Col1a1*, collagen type I α1 chain; *Spp1*, secreted phosphoprotein 1. Data are expressed as means ± SEM, and significance was determined by 2-way ANOVA and post hoc Tukey’s or Šidák’s multiple-comparison tests. ^#^*P* < 0.05 for WT vs. Adn-*Lpin1^–/–^* and †*P* < 0.05 for HSLF vs. HFHF-C diet (*n* = 7–9).

Although Adn-*Lpin1^–/–^* mice exhibited transcriptional profiles consistent with the development of MASH, there were no significant changes in plasma alanine transaminase (ALT) or aspartate aminotransferase (AST) levels, surrogate markers of liver damage, and histological scoring of H&E liver sections did not reveal any significant changes (Supplemental Figure 9, B and C). Because mice are inherently resistant to developing MASH on standard high-fat diets (14), we used a diet high in fructose (17 kcal %), fat (mostly palm oil, 40 kcal %), and cholesterol (2%) (HFHF-C) or a high-sugar (dextrose), low-fat (10 kcal % fat) control diet (HSLF) (Figure 8 and Supplemental Table 3). After 16 weeks, the HFHF-C diet did not affect body weight, blood glucose, or plasma insulin concentrations compared with the HSLF diet in each respective genotype (Figure 8A and Supplemental Figure 10A). However, Adn-*Lpin1^–/–^* mice were leaner on both diets compared with WT mice (Figure 8, A–D). Analogously to the short-term HFD studies (Supplemental Figure 3), Adn-*Lpin1^–/–^* mice had similar changes in plasma concentrations of NEFA, glycerol, and TAG, while cholesterol was increased in comparison with WT mice (Supplemental Figure 10B). The livers of Adn-*Lpin1^–/–^* mice were larger compared with controls, reaching about 13% of body weight on the HFHF-C diet (Figure 8E). Adn-*Lpin1^–/–^* mice also had enlarged spleens and significant elevations in plasma ALT, but not AST, on the HFHF-C diet, indicating increased liver injury (Figure 8, F–H). There was no significant difference in hepatic TAG content between genotypes and only trends toward an increase in metabolic dysfunction–associated steatotic liver disease (MASLD) scoring (Figure 8, I–L). However, gene expression markers of hepatic stellate cell activation and inflammation were increased in Adn-*Lpin1^–/–^* compared with WT control mice on HFHF-C diet (Figure 8, M–O). These data indicate that the loss of adipocyte *Lpin1* exacerbates liver injury and activation of markers of MASH in mice.

## Discussion

Dysfunctional adipose tissue may play a critical role in the development of metabolic abnormalities and diseases, including insulin resistance and MASLD. But the mechanisms by which this occurs remain incompletely understood. Herein, we have shown that adipocyte *LPIN1* expression is reduced in people with obesity compared with people who are lean. We found that *LPIN1* expression correlates with measures of skeletal muscle and hepatic insulin sensitivity and inversely correlates with hepatic de novo lipogenesis (DNL), a sensitive marker of hepatic insulin resistance and a characteristic of MASLD (11). In mice, the loss of *Lpin1* in adipocytes leads to a lean phenotype but promotes adipose tissue dysfunction and negatively affects systemic metabolism. Dysfunctional adipose tissue can be defined by a variety of qualities, including diminished capacity for lipid storage, insulin resistance, increased inflammation and fibrosis, and altered secretion of adipokines and other factors (15–18). Deletion of *Lpin1* in adipocytes impacted each of these parameters, as we have observed reduced rates of TAG synthesis (4), signs of adipose tissue inflammation and fibrosis, and reduced secretion of the beneficial adipokine adiponectin. Our indirect calorimetry data did not reveal any inherent differences in energy homeostasis or energy intake that would explain the differences in body weights between genotypes. However, these studies were conducted in adult mice over a period of 8 days and do not reflect the lifetime of metabolic adaptations to the loss of *Lpin1* early in life. Further research using inducible models of *Lpin1* ablation are required to better delineate the mechanisms suggested here.

Although Adn-*Lpin1^–/–^* mice were lean, they showed features of insulin resistance and steatosis observed in our participants with obesity. A recent study suggests that increased BMI and visceral adiposity are associated with increased adipose tissue inflammation and MASLD, even in lean subjects, and track with the severity of liver disease (14). These data are consistent with the idea that systemic influences of adipose tissue on metabolism are based on quality rather than quantity of adipose tissue. A caveat to our studies is that MUO is an acquired disease, and congenital deletion of lipin 1 in mice may not mimic what occurs in people. However, patients with lipodystrophy (both acquired and inherited forms) present with comorbidities similar to those seen in patients with MUO, including insulin resistance and MASLD (19, 20). Previous studies have used a conditional allele to knock out *Lpin1* in adult adipocytes, but this was done in lean chow-fed mice without obesity (5). Exactly how and when adipose tissue *Lpin1* expression declines in obesity remains controversial, as studies have reported both increased and decreased adipose tissue lipin 1 mRNA in obesity, which may be dependent on diet/genetic model and duration of obesity (4, 5, 21, 22).

One of our most striking observations was the phenotype of increased hepatic insulin resistance and lipid accumulation (Figures 4 and 5) and the demonstration that loss of adipocyte *Lpin1* is sufficient to drive liver injury in mice fed a MASH-inducing diet (Figure 7). Bulk RNA sequencing of liver tissue and WGCNA analysis revealed that Adn-*Lpin1^–/–^* mice exhibit dysregulation of several molecular signaling pathways known to impact the development of MASH (yellow module, Figure 6 and Supplemental Figure 7). Moreover, the accumulated intrahepatic DAG/TAG species correlate to increased expression of genes and genetic pathways associated with steatosis-to-MASH transition, including *Col1a1* and cell adhesion/ECM remodeling pathways (Figures 6 and 7 and Supplemental Table 2). The results from our WGCNA analysis reveal strong correlations between DAG/TAG species and these disease pathways (Figures 6 and 7). Species with long-chain unsaturated fatty acyl chains had the highest correlation with pathways associated with redox state, fatty acid metabolism, and MASH (turquoise and pink modules, Figure 6 and Supplemental Figure 7). In contrast, on the MASH-inducing diet, the enhanced liver injury exhibited by Adn-*Lpin1^–/–^* mice was not accompanied by increased intrahepatic TAG content or hyperinsulinemia. This suggests that other factors, including lower circulating adiponectin levels, could also be involved.

Ectopic lipid accumulation in insulin target tissues is tightly linked to the development of hepatic and systemic insulin resistance. In particular, tissue DAG accumulation has been mechanistically linked to the development of liver and skeletal muscle insulin resistance (23). Prior work suggested that mice lacking lipin 1 in adipose tissue were insulin resistant (5, 9), but the specific tissues that are impacted had not been investigated. Hyperinsulinemic clamp studies of HFD-fed Adn-*Lpin1^–/–^* mice revealed increased rates of glucose appearance from liver and showed that insulin failed to suppress hepatic glucose production. Clamp studies also demonstrated that Adn-*Lpin1^–/–^* mice exhibited reduced glucose uptake into skeletal muscle and BAT, suggesting that loss of lipin 1 in adipose tissue leads to multi-tissue insulin resistance. Although gastrocnemius DAG levels were elevated in the Adn-*Lpin1^–/–^* mice, future studies are required to address the specific causes of systemic insulin resistance in mice lacking adipocyte lipin 1.

The sources of the lipids accumulating in liver of Adn-*Lpin1^–/–^* mice remain to be determined. In this work and prior studies (4), we show that plasma NEFA and glycerol concentrations are actually lower in adipocyte-specific *Lpin1-*knockout mice (Supplemental Figure 3, A and B), suggesting that the hepatic lipids are not derived from increased lipolysis. Indeed, we have previously shown that loss of lipin 1 in adipocytes leads to reduced rates of lipolysis due to suppression of protein kinase A signaling (4). In mice fed the HFDs, hepatic lipids could be derived from the uptake of dietary lipids, but increased DAG and TAG were also observed in Adn-*Lpin1^–/–^* mice on diets with low fat content. In people with obesity and MASLD, intrahepatic DNL is increased several-fold compared with that in lean subjects and contributes to intrahepatic and plasma TAG (11, 24). We also observed increased fractional synthesis rates of palmitate in Adn-*Lpin1^–/–^* mice, consistent with our finding in human subjects of an inverse correlation between adipose tissue Adn-*Lpin1^–/–^* gene expression and hepatic DNL. WGCNA analysis also revealed that DNL genes clustered to the purple module, which was positively associated with the Adn-*Lpin1^–/–^* mice and monounsaturated fatty acyl chains (Figure 7). It is likely that increased rates of DNL observed in these mice, which may be driven by their insulin resistance, contribute to the observed hepatic steatosis even on the LFD.

In summary, the present study bolsters the argument that adipose tissue *function* rather than quantity is critical for preserving metabolic health. This work unveiled a strong correlation between adipose tissue *LPIN1* expression and hepatic and skeletal muscle insulin sensitivity as well as an inverse correlation with hepatic DNL in people. These findings provide evidence that loss of adipocyte lipin 1 is sufficient to induce systemic metabolic perturbations in mice. The mechanisms of inter-organ communication involved in these phenotypes are likely multifactorial. Future work is needed to define which factors play a causal role in the inter-organ communication between adipose tissue and other important insulin target tissues.

## Methods

### Sex as a biological variant

Our studies included male and female mice and yielded similar results within each sex. Sex differences were not determined because the male and female studies were conducted at different times and using different litters. Our human studies examined male and female patients and considered sex as a variable in all calculations and results.

### Human studies

#### Study subjects.

Some of the data reported here were obtained from a subset of subjects as part of their participation in other studies (11, 25). Sixty-one men and women participated in this study. All participants completed a comprehensive screening evaluation, including a medical history and physical examination, standard blood tests, hemoglobin A_1c_ (HbA1c), an oral glucose tolerance test (OGTT), and assessment of intrahepatic triglyceride (IHTG) content using MRI to determine eligibility. Subjects were characterized by body weight status and metabolic health into 3 groups: (a) metabolically healthy lean (MHL; *n* = 14, 8 women), defined as BMI 18.5–24.9 kg/m^2^, IHTG content <5%, plasma TAG concentration <150 mg/dL, normal fasting plasma glucose (<100 mg/dL), normal oral glucose tolerance (plasma glucose, <140 mg/dL 2 hours after ingestion of 75 g of glucose), and HbA1c ≤5.6%; (b) metabolically healthy obese (MHO; *n* = 25, 21 women), defined as BMI 30–49.9 kg/m^2^ with normal IHTG content, plasma TAG, and glucose tolerance; and (c) metabolically unhealthy obese (MUO; *n* = 25, 19 women), defined as BMI 30–49.9 kg/m^2^ with IHTG content ≥5.6%, and abnormal glucose metabolism determined by HbA1c 5.7%–6.4%, fasting plasma glucose 100–125 mg/dL, or 2-hour OGTT plasma glucose concentration 140–199 mg/dL. Potential participants who had a history of diabetes or liver disease other than MASLD, consumed excessive amounts of alcohol (>21 units per week for men and >14 units per week for women), or were taking medications that could affect the study outcome measures that precluded MRI were excluded from the study. Additional subject characteristics are listed in Supplemental Table 1.

#### Body composition analyses.

Total body fat and fat-free mass were determined by dual-energy x-ray absorptiometry. Intra-abdominal and subcutaneous abdominal adipose tissue volumes and IHTG content were assessed by MRI.

#### Insulin sensitivity and adipose tissue biopsy.

Subjects were admitted to the Clinical and Translational Research Unit at Washington University School of Medicine in St. Louis, Missouri, at 1700 hours for approximately 48 hours and consumed standard meals (50% carbohydrate, 35% fat, 15% protein) containing one-third of their estimated energy requirements (26) at 1800 hours on day 1 and at 0700 hours, 1300 hours, and 1900 hours on day 2. After the evening meal was consumed on day 2, participants fasted until the end of the hyperinsulinemic-euglycemic clamp procedure conducted on day 3. At 0700 hours on day 3, a primed (8.0 μmol/kg) continuous (0.08 μmol/kg/min) infusion of [U-^13^C]glucose (Cambridge Isotope Laboratories Inc.) was started through an intravenous catheter inserted into an antecubital vein. An additional catheter was inserted into a radial artery to obtain arterial blood samples. After infusion of glucose tracer for 210 minutes (basal period), insulin was infused for 210 minutes at a rate of 50 mU/m^2^ body surface area (BSA)/min (initiated with a 2-step priming dose of 200 mU/m^2^ BSA/min for 5 minutes followed by 100 mU/m^2^ BSA/min for 5 minutes). The infusion of [U-^13^C]glucose was stopped during insulin infusion because of the expected decrease in hepatic glucose production (27). Euglycemia (~100 mg/dL) was maintained by variable infusion of 20% dextrose enriched to approximately 1% with [U-^13^C]glucose. Blood samples were obtained before the beginning of the tracer infusion and every 6–7 minutes during the final 20 minutes (total of 4 blood samples) of the basal and insulin infusion periods. Abdominal subcutaneous adipose tissue was obtained during the basal stage of the hyperinsulinemic-euglycemic clamp procedure from the periumbilical area by aspiration through a 3 mm liposuction cannula (Tulip Medical Products) connected to a 30 cc syringe. Samples were immediately rinsed in ice-cold saline and frozen in liquid nitrogen before being stored at –80°C until final analyses.

#### Hepatic de novo lipogenesis.

Subjects consumed 50-mL aliquots of 70% D_2_O (Sigma-Aldrich), provided in sterile vials, every day for 3–5 weeks. Aliquots of D_2_O were consumed 3–4 times per day every day for the first 5 days (priming period) followed by two 50-mL doses daily. A blood sample was obtained after an overnight fast the day after the final dose of D_2_O was consumed to determine body water D_2_O enrichment and to measure hepatic DNL by gas chromatography–mass spectrometry (GC-MS) (11, 28). Compliance with D_2_O consumption was monitored by interview at weekly visits with the study research coordinator, by counting of the return of empty vials at each visit, and by evaluation of D_2_O enrichments in plasma (obtained on day 7 and weekly thereafter) and saliva (obtained on days 2, 4, and 11 and weekly thereafter).

#### Adipose tissue RNA sequencing.

Total RNA was isolated from frozen adipose tissue samples using QIAzol and an RNeasy Mini Kit in combination with an RNase-free DNase Set (QIAGEN) (29). Library preparation of the samples was performed with mRNA reverse-transcribed to yield cDNA fragments, which were then sequenced by an Illumina NovaSeq 6000 at the UCSD Institute for Genomic Medicine Genomics Center. Expression of *LPIN1* is presented as log_2_-transformed counts per million reads. The human RNA sequencing data were deposited in the Gene Expression Omnibus database (GEO; https://www.ncbi.nlm.nih.gov/geo; accession no. GSE156906).

#### Sample analysis and calculations.

Plasma glucose concentration was determined by an automated glucose analyzer. Plasma insulin, HbA1c, and lipid profile were measured in the Washington University Core Laboratory for Clinical Studies. Deuterium enrichment in total body water, deuterium enrichment and labeling pattern in TAG-palmitate, and [U-^13^C]glucose enrichment in plasma glucose were determined by GC-MS as described previously (30, 31). The hepatic insulin sensitivity index (HISI) was calculated as the inverse of the product of plasma insulin concentration and the endogenous glucose rate of appearance (Ra) in the systemic circulation, determined by division of the glucose tracer infusion rate by the average plasma glucose tracer-to-tracee ratio during the last 20 minutes of the basal period of the hyperinsulinemic-euglycemic clamp procedure (HECP) (3). The total glucose rate of disappearance (Rd) during insulin infusion was assumed to be equal to the sum of the endogenous glucose Ra and the rate of infused glucose during the last 20 minutes of the HECP (3). An index of whole-body insulin sensitivity was calculated as glucose Rd per kilogram fat-free mass divided by plasma insulin during the hyperinsulinemic-euglycemic clamp procedure. The fractional contribution of DNL to palmitate in plasma TAG was calculated by mass isotopomer distribution analysis as described previously (28, 30).

### Mouse models

Male and female mice were maintained in the C57BL/6J background. Mice were group-housed, given free access to a standard chow diet unless otherwise stated, and maintained on a standard 12-hour light/12-hour dark cycle. Mice harboring an *Lpin1*-floxed allele were previously described [B6(Cg)-*Lpin1^tm1c(EUCOMM)HMGU^*/FincJ; available from The Jackson Laboratory, strain 03211] (6). Adipocyte-specific knockout mice were generated by crossing of *Lpin1*-floxed mice with mice expressing adiponectin promoter–driven Cre recombinase [B6;FVB-Tg(Adipoq-cre)1Evdr/J; The Jackson Laboratory, strain 028020]. Littermate control mice were homozygous for *Lpin1-*floxed alleles but did not express Cre recombinase.

Before sacrifice, all mice were fasted for 4 hours starting at 0900. Mice were euthanized via CO_2_ asphyxiation. Blood was procured from the inferior vena cava into EDTA-coated tubes, and plasma was separated via centrifugation. All tissues were excised and immediately flash-frozen in liquid nitrogen and stored at –80°C until further use.

#### Diet studies.

Male mice were given ad libitum access to the indicated diets at 8 weeks of age. A high-fat diet (HFD; 60 kcal %, Research Diets, D12492) or a matched sucrose low-fat diet (LFD; 10 kcal %, Research Diets, D12450J) was provided for 5 weeks. For the MASH studies, a diet high in fructose (17 kcal %), fat (palm oil, 40 kcal %), and cholesterol (2%) (HFHF-C; Research Diets, D09100310) or a matched sucrose, high-sugar (dextrose), low-fat (10 kcal %) diet (HSLF; Research Diets, D09100304) was provided for 16 weeks. Specific nutrient composition and further details about percentage of total kilocalories in diet (%kcal) are listed in Supplemental Table 3.

#### Glucose, insulin, and olive oil tolerance tests.

Before all tolerance tests, mice were placed on hardwood bedding, and fasting was initiated at 0900 hours. Glucose tolerance tests were performed in 5-hour-fasted mice. Glucose was dissolved in saline and given via an intraperitoneal injection (1 g glucose/kg lean mass). Insulin tolerance tests were performed in 4-hour-fasted mice. Recombinant human insulin (Humalin R, Eli Lilly; 0.75 U/kg lean mass) in saline was given via an intraperitoneal injection. Blood was procured from the tail vein, and glucose was monitored via a glucometer (One Touch Ultra, Life Scan Inc.) at the times indicated. Lean mass and fat mass were determined using and EchoMRI before tolerance testing to determine lean mass for injections. Mice were fasted 4 hours before olive oil gavage (200 μL). Blood was collected from the tail vein at the times indicated. Plasma was separated and TAGs were measured using an enzymatic assay according to the manufacturer’s instructions (Thermo Fisher Scientific, TR22421).

#### Mouse hyperinsulinemic-euglycemic clamp studies.

Hyperinsulinemic-euglycemic clamp studies were performed at the Vanderbilt Mouse Metabolic Phenotyping Center. Catheters were implanted into a carotid artery and a jugular vein of mice for sampling and infusions, respectively, 5 days before the study as described by Berglund et al. (32). Insulin clamps were performed on mice fasted for 5 hours using a modification of the method described by Ayala et al (33). [3-^3^H]Glucose was primed (1.5 μCi) and continuously infused for a 90-minute equilibration and basal sampling periods (0.075 μCi/min). [3-^3^H]Glucose was mixed with the non-radioactive glucose infusate (infusate-specific activity of 0.5 μCi/mg) during the 2-hour clamp period. Arterial glucose was clamped using a variable rate of glucose (plus trace [3-^3^H]glucose) infusion, which was adjusted based on the measurement of blood glucose at 10-minute intervals. By mixing of radioactive glucose with the non-radioactive glucose infused during a clamp, deviations in arterial glucose-specific activity are minimized and steady-state conditions are achieved. The calculation of glucose kinetics is therefore more robust (34). Baseline blood or plasma variables were calculated as the mean of values obtained in blood samples collected at –15 and –5 minutes. At time zero, insulin infusion (4 mU/kg body weight/min) was started and continued for 120 minutes. Mice received heparinized saline-washed erythrocytes from donors at 5 μL/min to prevent a fall in hematocrit. Blood was taken from 80 to 120 minutes for the determination of [3-^3^H]glucose. Clamp insulin was determined at *t* = 100 and 120 minutes. At 120 minutes, 13 μCi of 2-[^14^C]deoxyglucose ([^14^C]2DG) was administered as an intravenous bolus. Blood was taken from 2 to 25 minutes for determination of [^14^C]2DG. After the last sample, mice were anesthetized and tissues were freeze-clamped for further analysis. Plasma insulin was determined by radioimmunoassay, and plasma NEFA and glycerol were measured enzymatically as described below. Analytes were measured at 10 minutes before clamp (Fasting) and averaged at 90 and 120 minutes after the start of the clamp (Clamp). Radioactivity of [3-^3^H]glucose and [^14^C]2DG in plasma samples and [^14^C]2DG-6-phosphate in tissue samples was determined by liquid scintillation counting. Glucose appearance (Ra) and disappearance (Rd) rates were determined using steady-state equations (35). Endogenous Ra was determined by subtraction of the glucose infusion rate from total Ra. The glucose metabolic index (Rg) was calculated as previously described (36).

#### Indirect calorimetry and metabolic studies.

Indirect calorimetry studies were conducted at the Kansas Center for Metabolism and Obesity Research as previously described (37). Eight-week-old male mice were fed ab libitum an LFD (*n* = 5–8) or HFD (*n* = 7–10) for 5 weeks with a standard 12-hour light at room temperature. Two weeks before indirect calorimetry, mice were acclimated to individual housing. After 5 weeks of diet, energy metabolism was assessed for 8 days in a Promethion continuous metabolic monitoring system (Sable Systems International). Energy intake was based on food weight changes per cage over the 8-day period and calculated based on energy density of the diets (Research Diets; Supplemental Table 3). Mice were acclimated 5 days before metabolic measurements that included total energy expenditure (EE; kcal/24 h), resting EE (kcal/24 h), non-resting EE (kcal/24 h), respiratory quotient (VCO_2_/VO_2_), and spontaneous cage activity (*X*, *Y*, and *Z* beam breaks). EE per 24 hours was calculated by a modified Weir equation as previously described (37). Total EE was calculated as the average EE for a 24-hour period over the last 2–3 days. Resting EE was calculated as the EE (kcal/24 h) during the 30-minute period with the lowest daily EE (kcal/h) times 24, and averaged across the last 2–3 days. Non-resting EE was calculated as total EE minus resting EE (37). Cage activity was calculated as beam breaks across 3 planes (*X* + *Y* + *Z*), and meters that the mouse moved were the summed distances calculated from Pythagoras’ theorem based on *XY* second-by-second coordinates. ANCOVA statistical analysis of the impact of the difference in body weight and fat mass on EE was not possible, as the data failed the homogeneity of regression test.

#### Histology and MASLD scoring.

Tissues were harvested as described above. Tissues were fixed in 10% neutral-buffered formalin for 48 hours. Samples were rinsed and stored in 70% EtOH until paraffin embedding, sectioning, and staining with H&E in the Anatomic Molecular Pathology Core Labs at Washington University School of Medicine. MASLD scoring was conducted by a blinded independent clinical pathologist, and the MASLD total score (*x*/8) was a summation of the steatosis score (*x*/3), lobular inflammation (*x*/3), and ballooning (*x*/2) (38).

#### Immunoblotting.

Thirty micrograms of protein was loaded onto 4%–15% acrylamide precast gels (Bio-Rad, 64329760) in Laemmli loading buffer (Bio-Rad, 161-0737). Proteins were transferred to PVDF membranes in Tris-glycine buffer with 10% methanol. After transfer, membranes were blocked in 5% BSA in TBS for 1 hour before overnight antibody incubations in 5% BSA in TBS. Antibodies used were as follows: lipin 1 (rabbit, Santa Cruz Biotechnology, sc-98450), lipin 2 (rabbit, as previously described, ref. 39), and β-actin (mouse, Sigma-Aldrich, SAB4502543). After incubations, membranes were washed and incubated in secondary antibodies (LI-COR) before imaging on a LI-COR Odyssey.

#### Liver triglyceride and plasma analyses.

Flash-frozen liver tissue was thawed and homogenized in ice-cold PBS (100 mg/mL). Lipids were solubilized in 1% sodium deoxycholate via vortexing and heating at 37°C for 5 minutes. TAGs were determined enzymatically using the Infinity triglyceride colorimetric assay according to the manufacturer’s instructions (Thermo Fisher Scientific, TR22421). Blood from 4-hour-fasted mice was collected into EDTA-coated tubes. Plasma was separated via centrifugation at 8,000*g* for 8 minutes at 4°C. Plasma lipids were determined using commercially available colorimetric assays as follows: triglycerides (Thermo Fisher Scientific, TR22421), non-esterified free fatty acids (NEFAs; Wako, 999-34691, 995-34791, 991-34891, and 993-35191), and free glycerol (Sigma-Aldrich, F26248). Plasma alanine transaminase (ALT) and aspartate aminotransferase (AST) were measured using liquid kinetic assays (TECO Diagnostic, A534 and A559). Plasma insulin was determined by Single Plex Immunoassay at the Washington University Core Laboratory for Clinical Studies. Plasma adiponectin and adipokines (leptin, resistin, and TNF-α) were determined by the Cellular Molecular Biology Core at the Washington University Nutrition Obesity Research Center using suspension magnetic bead–based Single Plex (adiponectin) or Multiplexed Immunoassays (MilliporeSigma, MADPNMAG-70K-01 and MADKMAG-71K) following the manufacturer’s protocol and were analyzed using a Luminex 200 system (Luminex Corp.)

#### De novo hepatic lipogenesis.

Total liver palmitate was analyzed for mass isotope abundances by liquid chromatography–mass spectrometry, using mass isotopomer distribution analysis (MIDA) to determine the effective body water deuterium exposure (precursor pool enrichment) for the calculation of fractional DNL (11, 40, 41). A detailed description of the methods is available in Supplemental Methods.

#### Lipidomic analysis.

Tissue and plasma lipidomic analysis was performed at the Washington University Metabolomics Facility as previously described (42). The liver and gastrocnemius muscle samples were homogenized in water (4 mL/g tissue). All the lipids were extracted from 50 μL of plasma or homogenate using protein precipitation method. The DAG and TAG were further extracted with modified Bligh-Dyer method. DAG (21:0-21:0) (2 μg/sample) and TAG (17:1-17:1-17:1) (13 μg/sample) were used as internal standards in plasma. DAG (21:0-21:0) (0.3 μg/sample) and TAG (17:1-17:1-17:1) were used as internal standards in liver and gastric samples. Internal standards were added to the samples before extraction. Quality control (QC) samples were prepared by pooling of the aliquots of the study samples and were used to monitor the instrument stability. The QC was injected between every 5 study samples. Measurement of DAG was performed with a Shimadzu 20AD UFLC system coupled to an AB Sciex API4000 mass spectrometer operated in positive multiple reaction monitoring (MRM) mode. Measurement of TAG was performed with a Shimadzu 20AD UFLC system coupled to an AB Sciex 4000QTRAP mass spectrometer operated in positive MRM mode. Data processing was conducted with Analyst 1.6.3 (Applied Biosystems). The relative quantification of lipids is provided, and the data are reported as the peak area ratios of the analytes to the corresponding internal standards. The lipid species showed coefficient of variation (CV) less than 15% in QC sample injections.

#### mRNA isolation and quantification.

For mouse studies, total RNA was isolated from frozen tissue using TRIzol Plus RNA purification kits (Thermo Fisher Scientific, 12183555) according to the manufacturer’s instructions. Second, 2 mg of RNA was reverse-transcribed into cDNA using TaqMan High-Capacity reverse transcriptase (Life Technologies, 43038228). Quantitative PCR was performed using Power SYBR Green (Applied Biosystems, 4367659) and measured using an ABI QuantStudio 3 sequence detection system (Applied Biosystems). Results were quantified using 2^–ΔΔCt^ and are shown as relative expression with respect to the control groups. Primer sequences are listed in Supplemental Table 4.

#### Bulk RNA sequencing and WGCNA analysis.

Bulk RNA sequencing was performed at the Genomic Technologies and Access Center at the McDonald Genomic Institute of Washington University School of Medicine. Complete methodology can be found in Supplemental Methods. The mouse RNA sequencing data were deposited in the GEO database (https://www.ncbi.nlm.nih.gov/geo; accession no. GSE239490).

### Statistics

Data are reported as means ± SEM unless otherwise noted. *P* ≤ 0.05 was considered statistically significant.

For studies conducted in human subjects, statistical analyses were performed using SPSS (version 28) (IBM). One-way ANOVA was used to compare subject characteristics and adipose tissue *LPIN1* expression among MHL, MHO, and MUO groups with Fisher’s least significant difference post hoc procedure used to identify significant mean differences where appropriate. Relationships between adipose tissue *LPIN1* expression and metabolic variables were evaluated by linear and nonlinear regression analysis with the best fit to the data reported.

Data obtained from studies conducted in mice were analyzed using GraphPad Prism software. Independent and paired Student’s *t* test and 2-way ANOVA were performed where appropriate. Secondary post hoc analysis found differences in groups using either Tukey’s or Šidák’s multiple comparisons where appropriate. Comparisons and replicate numbers are listed in each figure legend.

### Study approval

The human studies were approved by the Human Research Protection Office of Washington University School of Medicine in St. Louis, Missouri (ClinicalTrials.gov, NCT02706262). Participants were recruited using the Volunteers for Health database at Washington University School of Medicine and by advertising in the community. Written, informed consent was obtained from all participants before they were enrolled in this study.

All animal studies were approved by the Institution Animal Care and Use Committee of Washington University. All procedures required for the hyperinsulinemic-euglycemic clamp were approved by the Vanderbilt University Animal Care and Use Committee.

### Data availability

All data are available in the Supporting Data Values file and are available upon request. Additional data are available in public repositories (GEO database, https://www.ncbi.nlm.nih.gov/geo; accession nos. GSE156906 and GSE239490).

## Author contributions

AL, JMS, and AJL conducted most of the experiments, data collection, and analysis. DF, TMS, RK, SY, and MKR conducted animal experiments and collected data. HP and GJP contributed to experimental design, implementation, and data analysis for the de novo lipogenesis analysis in mice. MS, GIS, JY, MKH, and SK conducted and analyzed human studies and assisted with data interpretation related to this article. EMM, RK, SY, RLF, and JRB conducted and analyzed metabolic cage studies. MH conducted the blinded analysis of nonalcoholic fatty liver scoring and contributed intellectually to the article. BNF and AJL conceived the project, designed experiments, supervised projects, and wrote the manuscript. All authors contributed to the editing of the manuscript and read and approved the final version.

## Supplementary Material

Supplemental data

Unedited blot and gel images

Supporting data values

## Version 1 10/15/2024

In-Press Preview

## Version 2 12/02/2024

Electronic publication
