# Supplementary material for: Adipocyte lipin 1 expression associates with human metabolic health and regulates systemic metabolism in mice
Source: J Clin Invest. 2024 Oct 15;134(23):e169722. doi: 10.1172/JCI169722 (PMC11601902; doi:10.1172/JCI169722)
Supplement: Supplemental data [file jci-134-169722-s065.pdf]

Supplemental Methods

De novo hepatic lipogenesis

Animals from each group were labeled with an intraperitoneal injection of 100% ^2H_2O saline (35 µl/g body weight ~3.5 % of body weight, deuterium oxide, Sigma 151890) and provided with 8% ^2H_2O drinking water for the remainder of the study to maintain body ^2H_2O enrichments of approximately 5%. Livers were then homogenized in acetonitrile, methanol and water (2:2:1) for fatty acid solubilization and subsequent analysis by LC-MS to quantify the isotopic enrichment (M1, M2, M3, M4) due to the incorporation of deuterium from heavy water into palmitate. Specifically, a 2 µl aliquot containing the fatty acid metabolites was subjected to LC/MS analysis by using an Agilent 1290 Infinity II (LC) system coupled to an Agilent 6545 Quadrupole-Time-of-Flight (QTOF) mass spectrometer with a dual Agilent Jet Stream electrospray ionization source. Samples were separated on a SeQuant ZIC-pHILIC column (100 3 2.1 mm, 5 mm, polymer, Merck-Millipore) including a ZIC-pHILIC guard column (2.1 mm x 20 mm, 5 mm) To confirm only palmitate eluted at its retention time, samples were analyzed with higher resolution (120,000) on an Agilent ID-X Orbitrap. The column compartment temperature was maintained at 40C and the flow rate was set to 250 mL/min. The mobile phases consisted of A: 95% water, 5% acetonitrile, 20 mM ammonium bicarbonate, 0.1% ammonium hydroxide solution (25% ammonia in water), 2.5 mM medronic acid, and B: 95% acetonitrile, 5% water, 2.5 mM medronic acid. The following linear gradient was applied: 0 to 1 min, 90% B; 12 min, 35% B; 12.5 to 14.5 min, 25% B; 15 min, 90% B followed by a re-equilibration phase of 4 min at 400 mL/min and 2 min at 250 mL/min. Palmitate ions were monitored at a mass of 256.240, 257.244, 258.247, and 259.248, representing the parent M0 through M3 isotopes. Excess M1 and M2 enrichments were determined by subtraction of the natural abundance values in unlabeled standards (run in parallel). The proportion of plasma palmitate that originated from the DNL pathway was then calculated from the excess M1 and M2 of palmitate by using MIDA to

determine both the biosynthetic precursor enrichment and the corresponding isotopic enrichment of newly synthesized palmitate molecules(1). The precursor pool enrichment (p) was determined from the ratio of EM2/EM1 in the experimental data. Knowledge of the calculated metabolic precursor pool enrichment and the known n (number of repeating subunits in the polymer = 21 for palmitate(2) allowed calculation of the theoretical asymptote enrichment of the single-labeled mass isotopomer species (excess M1), representing the maximum possible enrichment when palmitate is newly synthesized at this deuterium precursor pool enrichment.

Bulk RNA sequencing and WGCNA analysis

Samples were prepared according to library kit manufacturer's protocol, indexed, pooled, and sequenced on an Illumina HiSeq. Basecalls and demultiplexing were performed with Illumina's bcl2fastq software and a custom python demultiplexing program with a maximum of one mismatch in the indexing read. RNA-seq reads were then aligned to the Ensembl release 76 primary assembly with STAR version 2.5.1a (3). Gene counts were derived from the number of uniquely aligned unambiguous reads by Subread:featureCount version 1.4.6-p5 (4). Isoform expression of known Ensembl transcripts were estimated with Salmon version 0.8.2 (5). Sequencing performance was assessed for the total number of aligned reads, total number of uniquely aligned reads, and features detected. The ribosomal fraction, known junction saturation, and read distribution over known gene models were quantified with RSeQC version 2.6.2 (6).

All gene counts were then imported into the R/Bioconductor package EdgeR (7) and TMM normalization size factors were calculated to adjust for samples for differences in library size. Ribosomal genes and genes not expressed in the smallest group size minus one samples greater than one count per million were excluded from further analysis. The TMM size factors and the matrix of counts were then imported into the R/Bioconductor package Limma (8). Weighted likelihoods based on the observed mean-variance relationship of every gene and

sample were then calculated for all samples with the voomWithQualityWeights (9). The performance of all genes was assessed with plots of the residual standard deviation of every gene to their average log-count with a robustly fitted trend line of the residuals. Differential expression analysis was then performed to analyze for differences between conditions and the results were filtered for only those genes with Benjamini-Hochberg false-discovery rate adjusted *p*-values less than or equal to 0.05.

The heatmap was generated using iDEP 9.0 (10). For each contrast extracted with Limma, global perturbations in known Gene Ontology (GO) terms, MSigDb, and KEGG pathways were detected using the R/Bioconductor package GAGE(11) to test for changes in expression of the reported log 2 fold-changes reported by Limma in each term versus the background log 2 fold-changes of all genes found outside the respective term. Perturbed KEGG pathways where the observed log 2 fold-changes of genes within the term were significantly perturbed in a single-direction versus background or in any direction compared to other genes within a given term with p-values less than or equal to 0.05 were rendered as annotated KEGG graphs with the R/Bioconductor package Pathview (12).

To find the most critical genes, the raw counts were variance stabilized with the R/Bioconductor package DESeq2 (13) and was then analyzed via weighted gene correlation network analysis with the R/Bioconductor package WGCNA (14). Briefly, all genes were correlated across each other by Pearson correlations and clustered by expression similarity into unsigned modules using a power threshold empirically determined from the data. An eigengene was then created for each de novo cluster and its expression profile was then correlated across all coefficients of the model matrix. Because these clusters of genes were created by expression profile rather than known functional similarity, the clustered modules were given the names of random colors where grey is the only module that has any pre-existing definition of containing genes that do not cluster well with others. These de-novo clustered genes were then

tested for functional enrichment of known GO terms with hypergeometric tests available in the R/Bioconductor package clusterProfiler (15). Significant terms with Benjamini-Hochberg adjusted p-values less than 0.05 were then collapsed by similarity into clusterProfiler category network plots to display the most significant terms for each module of hub genes in order to interpolate the function of each significant module. The information for all clustered genes for each module were then combined with their respective statistical significance results from Limma to determine whether or not those features were also found to be significantly differentially expressed. Lipidomic and phenotypic data were tested for associations with WGCNA pathways using Pearson's Correlation Coefficient and significant correlations were considered if $p < 0.05$.

References

1. Hellerstein MK, Neese RA. Mass isotopomer distribution analysis at eight years: theoretical, analytic, and experimental considerations. *Am J Physiol*. 1999;276(6):E1146-1170.

2. Diraison F, Moulin P, Beylot M. Contribution of hepatic de novo lipogenesis and reesterification of plasma non esterified fatty acids to plasma triglyceride synthesis during non-alcoholic fatty liver disease. *Diabetes Metab*. 2003;29(5):478–485.

3. Dobin A, et al. STAR: ultrafast universal RNA-seq aligner. *Bioinformatics*. 2013;29(1):15–21.

4. Liao Y, Smyth GK, Shi W. featureCounts: an efficient general purpose program for assigning sequence reads to genomic features. *Bioinformatics*. 2014;30(7):923–930.

5. Patro R, et al. Salmon provides fast and bias-aware quantification of transcript expression. *Nature Methods*. 2017;14(4):417–419.

6. Wang L, Wang S, Li W. RSeQC: quality control of RNA-seq experiments. *Bioinformatics*. 2012;28(16):2184–2185.

7. Robinson MD, McCarthy DJ, Smyth GK. edgeR: a Bioconductor package for differential expression analysis of digital gene expression data. *Bioinformatics*. 2010;26(1):139–140.

8. Ritchie ME, et al. limma powers differential expression analyses for RNA-sequencing and microarray studies. *Nucleic Acids Res*. 2015;43(7):e47–e47.

9. Liu R, et al. Why weight? Modelling sample and observational level variability improves power in RNA-seq analyses. *Nucleic Acids Res*. 2015;43(15):e97.

10. Ge SX, Son EW, Yao R. iDEP: an integrated web application for differential expression and pathway analysis of RNA-Seq data. *BMC Bioinformatics*. 2018;19(1):534.

11. Luo W, et al. GAGE: generally applicable gene set enrichment for pathway analysis. *BMC Bioinformatics*. 2009;10(1):161.

12. Luo W, Brouwer C. Pathview: an R/Bioconductor package for pathway-based data integration and visualization. *Bioinformatics*. 2013;29(14):1830–1831.

13. Love MI, Huber W, Anders S. Moderated estimation of fold change and dispersion for RNA-seq data with DESeq2. *Genome Biol*. 2014;15(12):550.

14. Langfelder P, Horvath S. WGCNA: an R package for weighted correlation network analysis. *BMC Bioinformatics*. 2008;9(1):559.

15. Yu G, et al. clusterProfiler: an R Package for Comparing Biological Themes Among Gene Clusters. *OMICS: A Journal of Integrative Biology*. 2012;16(5):284–287.

Supplemental Data Figure 1: Adn-*Lpin1-/-* mice have a complete loss of lipin 1 in adipose tissue. Thirty-week-old chow-fed male mice were fasted for four hours prior to sacrifice and tissues collected. (**A**) gonadal white adipose tissue (gWAT), inguinal white adipose tissue

(iWAT), brown adipose tissue (BAT), liver, heart, and gastrocnemius muscle were were separated via electrophoresis and immunoblotted for lipin 1. Total protein is shown via Amido black staining. Eight-week-old male Adn-*Lpin1-/-* mice and their littermate controls (WT) were fed either a 10% low-fat diet (LFD) or a 60% high-fat diet (HFD) for 5 weeks. Mice were fasted for 4 hours prior to sacrifice and tissue collection. (**B**) Gonadal white adipose tissue (gWAT), inguinal white adipose tissue (iWAT), and liver tissue proteins were separated via electrophoresis and immunoblotted for lipin 1, lipin 2, and the loading control β-actin. (**C**) Gene expression was determined by qPCR and are expressed as relative abundance; lipin 1, lipin 2, and lipin 3 (*Lpin1, Lpin2, Lpin3).* Data are expressed as means +/- S.E.M, and significance was determined by Two-way ANOVA with post-hoc Tukey's multiple comparisons tests. $^{\#}p < 0.05$ for WT vs. Adn-*Lpin1-/-* and $^{\dagger}p < 0.05$ for LFD vs. HFD; (n = 7-8).

Supplemental Data Figure 2: Adn-*Lpin1-/-* mice are outwardly normal on a chow diet. Twelve-week-old male and female Adn-*Lpin1-/-* mice and their wild-type littermate controls (WT) were given ab libitum access to a standard chow diet. (**A**) Body weight of fed-male mice. (**B**) Body composition was determined by ECHO MRI. (**C** and **D**) Body weight and composition in female mice. (**E**-**H**) Insulin tolerance tests (ITT) were performed in mice fasted for 4 hours prior to an intraperitoneal (IP) injection of recombinant human insulin (0.75 U / kg lean mass) and blood glucose was monitored from tail blood at the times indicated. (Males n = 9-12), (Females n = 3-5). (**I** and **K**) Chow fed 12 week old male mice were fasted for 4 hours followed by an oral gavage of olive oil (200µl/ mouse). Blood was procured from the tail vein and measured for TAG content via colorimetric assay. Data are expressed as means +/- S.E.M. Significance was determined by Two-way ANOVA with post-hoc Tukey's multiple comparisons tests or Student's T-Test where appropriate. p values are shown.

Supplemental Data Figure 3: Loss of adipocyte *Lpin1* reduces plasma NEFA and adipokine concentrations. Eight-week-old male Adn-*Lpin1-/-* and control mice were fed either

a 10% LFD or a 60% HFD for 5 weeks. Mice were fasted for 4 hours prior to sacrifice and blood was collected into EDTA-coated tubes and plasma was separated via centrifugation. (**A**-**C**) Plasma non-esterified fatty acids (NEFA), glycerol, and triglycerides (TAG) were measured using colorimetric assays according to the manufacturers' instructions. (**D**) Plasma adiponectin was measured using a Singlex Immunoassay. (**E** and **F**) Plasma leptin and resistin were measured by Multiplex Immunoassays. Data are expressed as means +/- S.E.M, and significance was determined by Two-way ANOVA with post-hoc Tukey's multiple comparisons tests. $^{\#}p < 0.05$ for WT vs. Adn-*Lpin1-/-* and $^{\dagger}p < 0.05$ for LFD vs. HFD; (n = 7-9).

Supplemental Data Figure 4: Short-term HFD feeding causes insulin and glucose intolerance in Adn-*Lpin1-/-* mice. Eight-week-old male Adn-*Lpin1-/-* mice and their WT littermate controls were fed either a 10% low-fat diet (LFD) or a 60% high-fat diet (HFD) for 5 weeks. (**A**) During week 4 of dietary feeding, lean mass was determined via ECHO MRI, and mice were fasted for 4 hours prior to an insulin tolerance test (ITT) via an intraperitoneal (IP) injection of recombinant human insulin (0.75 U / kg lean mass). (**B**) After 5 weeks of diet mice were fasted 5 hours prior to a glucose tolerance test (GTT) via an IP injection of glucose (1 g/ kg lean mass, dissolved in saline. Blood glucose was monitored in tail blood at the times indicated. (**C**) Area under the curve was calculated for the GTT. (**E**) Gonadal, inguinal, brown adipose, liver, heart, and gastrocnemius muscle tissue proteins were separated via electrophoresis and immunoblotted for phospho-AKT, total AKT, phospho-GSK3β, total GSK3β, and the loading control, GAPDH. Data are expressed as means +/- S.E.M, and significance was determined by Two-way ANOVA with post-hoc Tukey's multiple comparisons tests. $^{\#}p < 0.05$ for WT vs. Adn-*Lpin1-/-* and $^{\dagger}p < 0.05$ for LFD vs. HFD; (n = 5-6).

Supplemental Data Figure 5: Bulk RNA sequencing in liver. Eight-week-old male Adn-*Lpin1-/-* and control mice were fed either a 10% LFD or a 60% HFD for 5 weeks. Mice were fasted for 4 hours prior to sacrifice, liver collection, RNA isolation, and Bulk RNA sequencing. (**A**) PCA plot

showing separation of the four groups. (**B**) Heatmap of merged differentially expressed data. (**C**) Volcano plots of merged differential expression data were graphed as $\log_2$ fold change versed –$\log_{10}$ unadjusted *p*-value. ($n = 6$).

Supplemental Data Figure 6: WGCNA module association with and phenotypic data and liver lipidomic data. WGCNA module sets were combined with phenotypic and lipidomic data and associations were tested using Pearson's Correlation Coefficient; $^*p < 0.05$, ($n = 6$). (**A**) WGCNA modules and their correlation to phenotypic traits from the mice used to generate the WGCNA data. (**B**) WGCNA modules associations with liver lipidomic data.

Supplemental Data Figure 7: Select WGCNA module set pathway analysis. a-c, Graphical representations of top KEGG, and GO pathways in the turquoise (**A**), pink (**B**) , yellow (**C**), cyan (**D**) and purple (**E**) module sets. The color of the circle represents its adjusted *p-value* and the size represents the number of genes altered within that pathway.

Supplemental data Figure 8: Metabolic gene expression and their modules. Eight-week-old male Adn-*Lpin1-/-* and control mice were fed either a 10% LFD or a 60% HFD for 5 weeks. Mice were fasted for 4 hours prior to sacrifice, liver collection, RNA isolation, and Bulk RNA sequencing. Genes are expressed as counts per million reads (CPM) fold change from WT LFD-fed mice. Each gene's module is represented in color. Data are expressed as means +/- S.E.M, and significance was determined by Two-way ANOVA with post-hoc Tukey's multiple comparisons tests. $^{\#}p < 0.05$ for WT vs. Adn-*Lpin1-/-* and $^{\dagger}p < 0.05$ for LFD vs. HFD; ($n = 6$).

Supplemental Data Figure 8: Loss of adipocyte *Lpin1* increases early signs of hepatic fibrosis, matrix remodeling, and inflammation. Eight-week-old male Adn-*Lpin1-/-* and control mice were fed either a 10% LFD or a 60% HFD for 5 weeks. Mice were fasted for 4 hours prior

to sacrifice. (**A**) Gene expression was determined by qPCR and are expressed as relative abundance; collagen type I alpha 1 chain (*Col1a1*), tissue inhibitor of metalloproteinase 1 & 3 (*Timp1 and Timp3*), secreted phosphoprotein 1 (*Spp1*), cluster of differentiation 68 (*Cd68*), interleukin 1 beta (*Il1b)*, transforming growth factor beta 1 (*TGFB1*). (**B**) Plasma alanine transferase (ALT) and aspartate aminotransferase (AST) were measured using liquid kinetic assays. (**C**) H&E stained tissue sections were scored by an independent clinical pathologist. Data are expressed as means ± S.E.M., and significance was determined by Two-way ANOVA with post-hoc Tukey's multiple comparisons tests. $^{\#}p < 0.05$ for WT vs. Adn-*Lpin1-/-*; (*n* = 5-9).

Supplemental Data Figure 10: Plasma characteristics of mice fed a NASH-inducing diet. Eight-week-old male mice were fed a diet high in fructose (17 kcal %), fat (palm oil 40 kcal %), and cholesterol (2%) (HFHF-C) or a matched high-sucrose low-fat (10 kcal %) control diet (HSHF) for 16 weeks. Mice were fasted for 4 hours prior to sacrifice and tissue collection. (**A**) Blood glucose and plasma insulin concentrations after a 4 hour fast. (**B**) Plasma non-esterified fatty acids (NEFA), glycerol, and triglycerides (TAG) were measured using colorimetric assays according to the manufacturers' instructions. (**C**) Plasma adiponectin was measured using a Singlex Immunoassay and plasma leptin and resistin were measured by Multiplex Immunoassays. Data are expressed as means +/- S.E.M, and significance was determined by Two-way ANOVA and post-hoc Tukey's or Sidak's multiple comparisons tests. $^{\#}p < 0.05$ for WT vs. Adn-*Lpin1-/-* and $^{\dagger}p < 0.05$ for HSLF vs HFHF-C diet; (*n* = 7-9).

Supplemental Data Table 1. Subject Characteristics.

	MHL (n=14)	MHO (n=22)	MUO (n=25)
Age (years)	36 ± 2	36 ± 2	41 ± 2
Age Range	27.1- 47.5	24.6- 47.5	22- 54.7
Sex (F/ M)	8/ 6	21/ 1	19/ 6
Black or African American / White Caucasian/ Asian	1/ 10/ 3	11/ 11/ 0	6/ 19/ 0
Body mass index (kg/m^2)	22.9 ± 0.4	37.8 ± 1.0*	39.3 ± 1.0*
Body fat (%)	29.4 ± 1.6	48.2 ± 1.1*	47.2 ± 1.5*
Intrahepatic triglyceride content (%)	1.8 ± 0.2	2.3 ± 0.2	17.9 ± 1.8*†
Plasma FFA (mM)	0.24 ± 0.01	0.23 ± 0.01	0.29 ± 0.01
Plasma TAG (mg/dL)	72 ± 8	70 ± 5	134 ± 9*†
Plasma total cholesterol (mg/dL)	177 ± 7	168 ± 6	180 ± 8
Plasma HDL (mg/dL	63 ± 4	54 ± 3	43 ± 2
Plasma LDL (mg/dL)	99 ± 6	100 ± 6	114 ± 6
Fasting Insulin (µM/mL)	5.2 ± 0.5	12.7 ± 1.6	28.4 ± 3.2
Fasting C-peptide (ng/mL)	1.52 ± 0.09	2.45 ± 0.14	4.37 ± 0.27
Fasting glucose (mg/dL)	86 ± 1	88 ± 1	103 ± 5*†
2-h OGTT glucose (mg/dL)	98 ± 5	108 ± 3	157 ± 4*†
HbA1c (%)	5.0 ± 0.2	5.1 ± 0.1	5.6 ± 0.1*†
HISI [1000/(umol/kg FFM/min) x (µU/mL)]	10.9 ± 1.2	5.7 ± 0.4*	3.0 ± 0.2*†
Glucose Rd/Insulin, (nmol/kg FFM/min)/(µU/mL)	683 ± 65	386 ± 37*	209 ± 17*†
Hepatic DNL (% contribution to plasma TG-palmitate)	14.0 ± 1.7	21.3 ± 2.1*	39.0 ± 2.4*†

Supplemental Data Table 2. Significantly upregulated genes and their WGCNA module association.

A LFD WT vs. Adn-*Lpin1* -/-

Gene	UP Log FC
Cidea	7.67
Ephb2	4.32
Ppp1r3g	3.79
Sprr1a	3.28
Ly6d	2.98
Col1a1	2.93
Gsta1	2.86
Cidec	2.77
Mogat1	2.77
Cyp2b10	2.68
Cdkn1a	2.51
Gprc5b	2.49
Apoa4	2.47
Gpnmb	2.46
Plin4	2.46
Fmod	2.45
Cyp4a14	2.38
Lgals1	2.35
Bglap3	2.35
Ntrk2	2.31
Cd36	2.30
Mmp12	2.28
Hr	2.27
Tmem119	2.24
Osbpl3	2.24
Themis	2.23
Ttc39a	2.22
Zfp979	2.22
Zfp979	2.22
Gm11695	2.22
Nr4a1	2.21
Cpxm1	2.17
A4gnt	2.14
Cntnap1	2.13
Pdk4	2.10
Mfap4	2.10
Dusp8	2.10
Rufy4	2.10
Fam180a	2.08
Gal3st1	2.07
Slc35f2	2.07
Dpep1	2.06
Nr4a2	2.05
Igfbp6	2.02
Zfp423	2.01

Gene	Down Log FC
Irx1	-2.07
Lcor	-2.41
Gprin3	-2.42
Obox4-ps2	-2.68
4930565N06Rik	-2.88
Mup-ps20	-2.99
Zfp871	-3.04
F830016B08Rik	-3.16
Moxd1	-3.19
1700023H06Rik	-3.27

B HFD WT vs. Adn-*Lpin1* -/-

Gene	UP Log FC
Cidea	8.15
Sprr1a	3.65
Cfd	3.60
B430212C06Rik	3.56
A4gnt	3.47
Gpnmb	3.34
Obp2a	3.30
Kbtbd11	3.01
Cidec	2.79
Ly6d	2.72
Mup9	2.66
Mogat1	2.60
Ephb2	2.57
Gsta1	2.46
Slc35f2	2.37
Mmp12	2.37
Limk1	2.28
Gtpbp4-ps1	2.25
Cyp2b9	2.21
Themis	2.18
Dusp8	2.18
Zfp979	2.16
Zfp979	2.16
Col1a1	2.14
Ttc39a	2.10
Plin4	2.06
Ttc39aos1	2.06
Apoa4	2.03

Gene	Down Log FC
4930565N06Rik	-2.14
Lnpep	-2.16
Obox4-ps2	-2.21
F830016B08Rik	-2.34
Gprin3	-2.37
Zfp871	-2.38
Lcor	-2.41
1700023H06Rik	-2.42
Adgrf1	-2.82
Tff3	-3.01
Tmeff2	-3.15
Capn11	-3.27

Color Key

# Genes	Module Colors
822	Yellow
2762	Turquoise
505	Red
273	Purple
355	Pink
128	Midnightblue
348	Magenta
612	Green
1120	Brown
1994	Blue
391	Black
244	Tan

C Adn-*Lpin1* -/- LFD vs. HFD

Gene	UP Log FC
Cyp2b9	5.94
Obp2a	3.11
Cfd	2.72
Cyp2b10	2.25
1810046K07Rik	2.15

Gene	Down Log FC
Capn11	-2.06
Adgrf1	-2.15
Pltp	-2.16
Lepr	-2.18
2310034O05Rik	-2.43
Moxd1	-2.46
Pnpla3	-2.60
Tff3	-2.83
Cib3	-3.03
Ppp1r3g	-3.13
Gm6166	-3.77
Gm14328	-4.00
Chrna4	-4.05
Fabp5	-4.37
Pnpla5	-4.91

D WT LFD vs. HFD

Gene	UP Log FC
Cyp2b9	4.35
Cyp2b10	3.71
Capn11	2.77
Igfbp6	2.49
Upk3b	2.11

Gene	Down Log FC
Chrna4	-2.04
4930452B06Rik	-2.09
Gm14236	-2.16
Pnpla3	-2.38
Mup-ps20	-2.54
Gm14328	-3.05
Fabp5	-3.24
Moxd1	-3.6
Gm6166	-3.71
Pnpla5	-3.8

Supplemental Data Table 3. Composition of diets used in the study.

**Name**	**LFD**	**HFD**	**HSLF**	**HFHF-C**
**Research Diets #**	**D12450J**	**D12492**	**D09100304**	**D09100310**
	% kcal	% kcal	% kcal	% kcal
Total Carbohydrate	70	20	70	40
Total Protein	20	20	20	20
Total Fat	10	60	10	40
Cholesterol (% weight)	-	-	-	2
Corn Starch	50.4	-	34.9	-
Dextrose, Monohydrate	-	-	16.8	-
Maltodetrin 10	12.4	12.4	8.5	10.0
Sucrose, Fine Granulated	6.8	6.8	9.6	9.6
Fructose	-	-	-	19.9
Casein, Lactic, 30 Mesh	19.9	19.9	19.9	19.9
Cystine, L	0.3	0.3	0.3	0.3
Lard	4.5	54.9	4.5	4.5
Soybean Oil, USP	5.6	5.6	5.6	5.6
Palm Oil	-	-	-	30.3
Cholesterol	-	-	-	0.0
Vitamin Mix	1.0	1.0	1.0	1.0

Supplemental Data Table 4: List of primer sequences used for qPCR.

Gene Name (Mouse)	Forward 5'-3'	Reverse 5'-3'
Lpin1	agtcagcatcgtatcccagttcg	aatctaccaggctgctgggg
Lpin2	gaagtggcggctctctatttc	agagggttacatcaggcaagt
Lpin3	tcacccttccacgtgcgcttc	tcttcctcactgtccagctcct
Pparg1	ggaagaccactcgcattcctt	gtaatcagcaaccattgggtca
AdipoQ	tgttcctcttaatcctgccca	ccaacctgcacaagttccctt
Col1a1	gctcctcttaggggccact	ccacgtctcaccattgggg
Tgfb1	ctcccgtggcttctagtgc	gccttagtttggacaggatctg
Cd68	agctgagggaagtgaatggaa	tgcctctttacacgggattgc
Timp1	gcaactcggacctggtcataa	cggcccgtgatgagaaact
Spp1	atctcaccattcggatgagtct	tgtagggacgattggagtgaaa
Il1B	gcaactgttcctgaactcaact	atcttttggggtccgtcaact

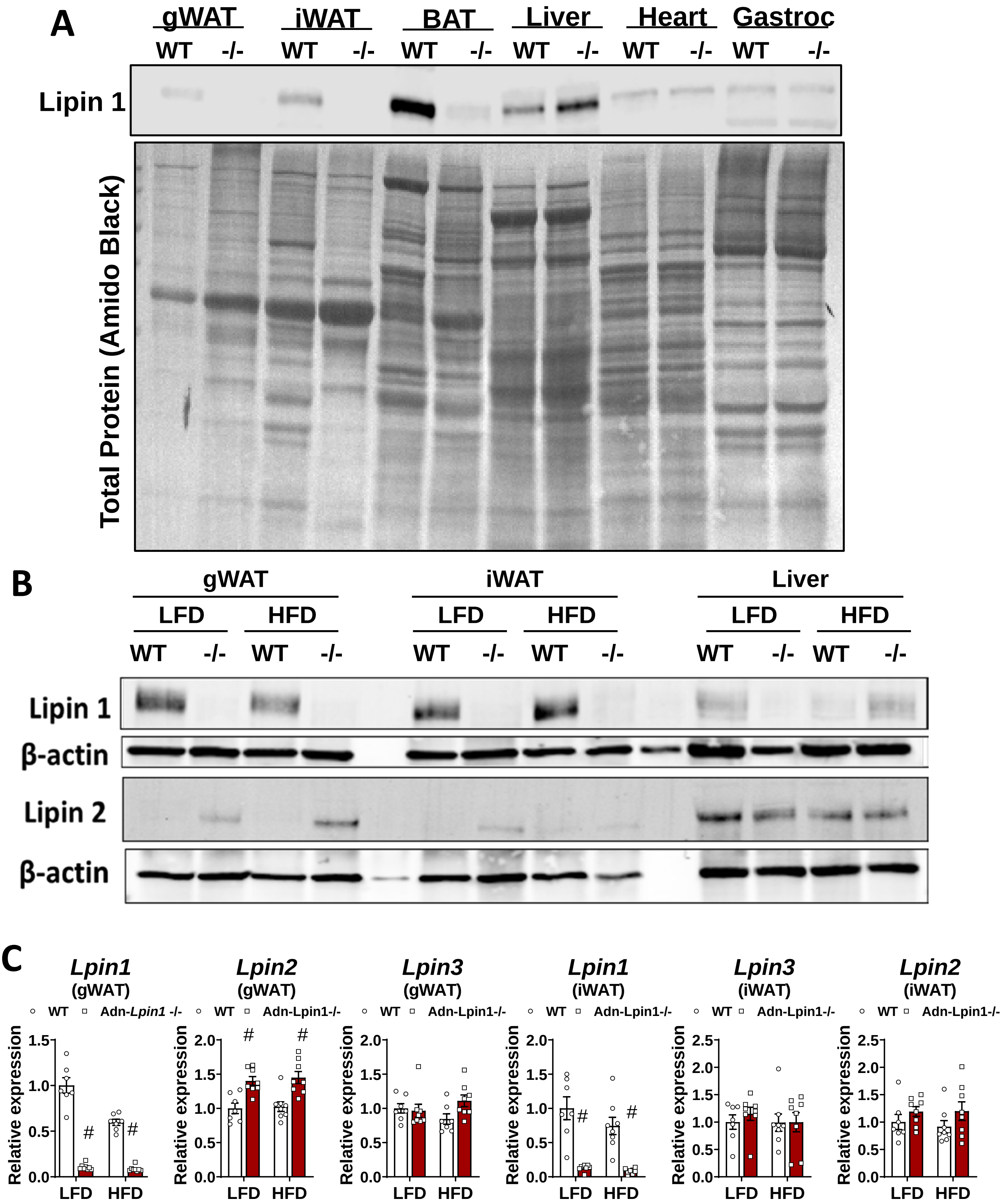

Supplemental Data Figure 1: Adn-*Lpin1-/-* mice have a complete loss of lipin 1 in adipose tissue.

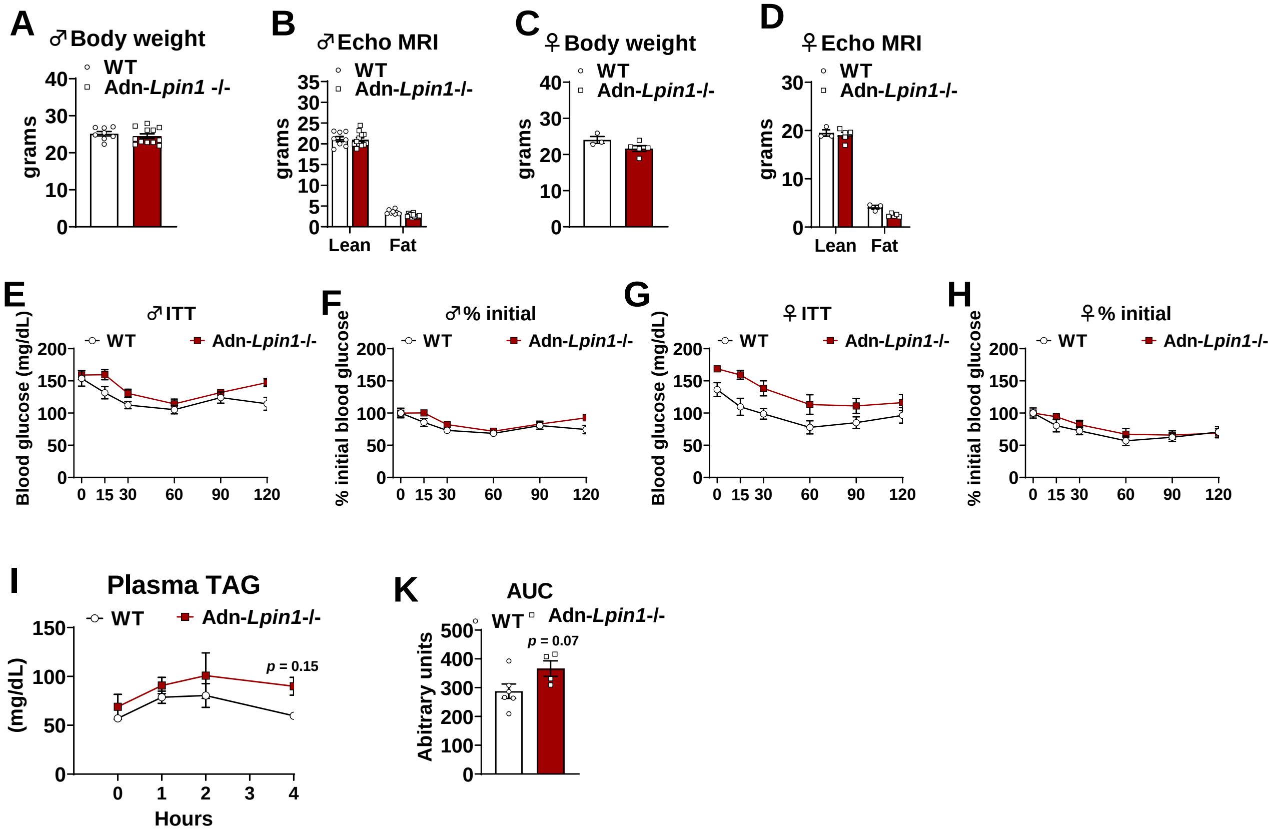

Supplemental Data Figure 2: Adn-*Lpin1-/-* mice are outwardly normal on a chow diet.

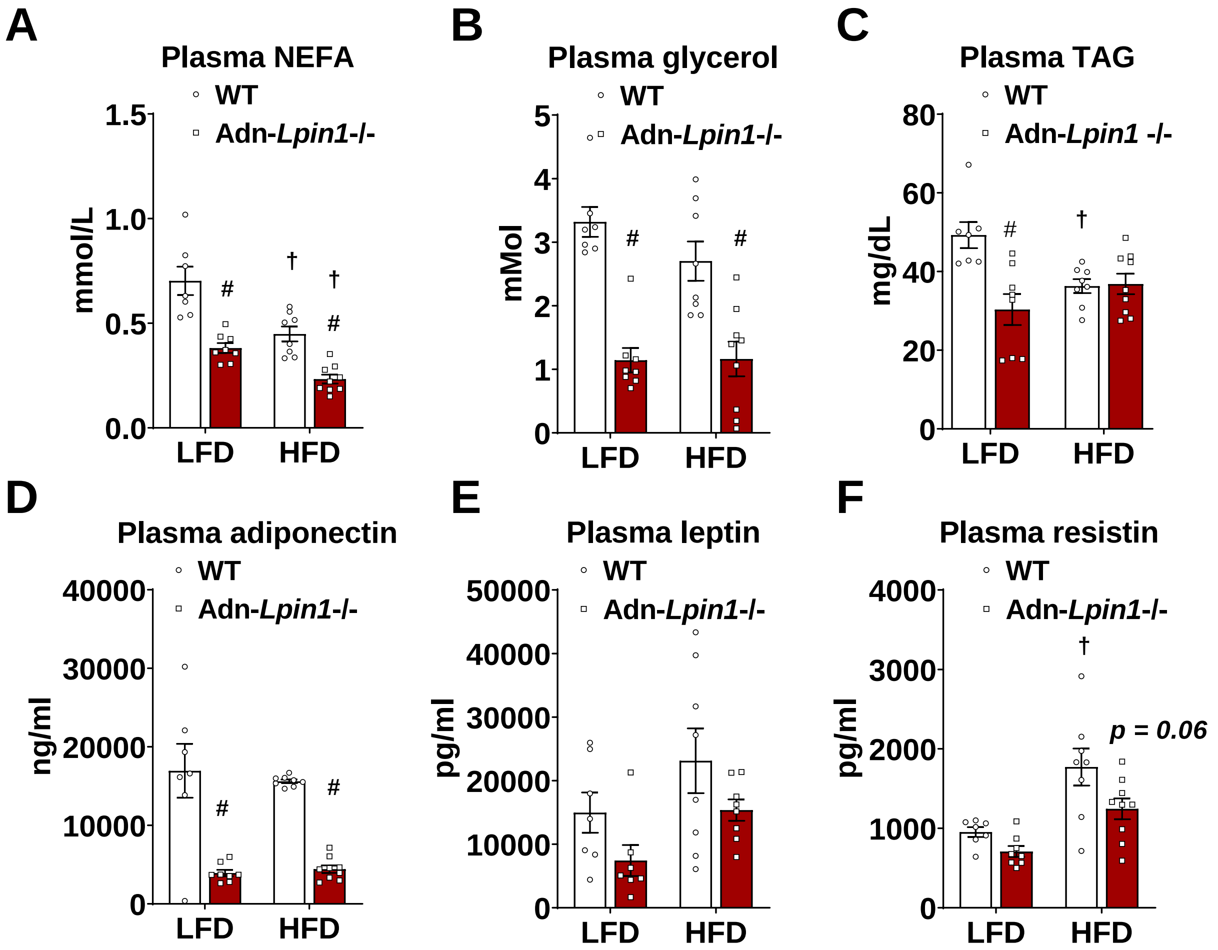

Supplemental Data Figure 3: Loss of adipocyte *Lpin1* reduces plasma NEFA and adipokine concentrations.

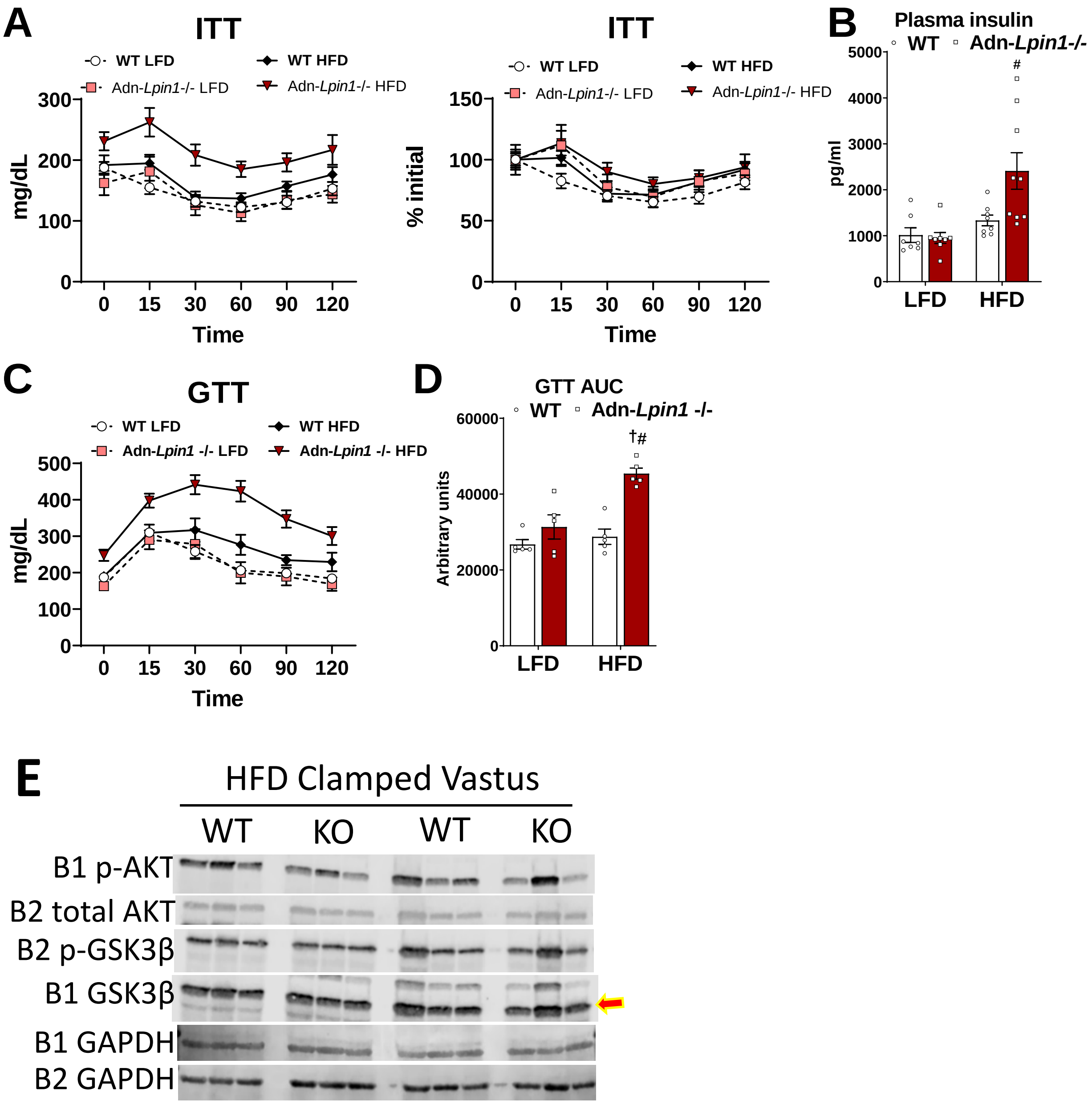

Supplemental Data Figure 4: Short-term HFD feeding causes insulin and glucose intolerance in Adn-*Lpin1-/-* mice.

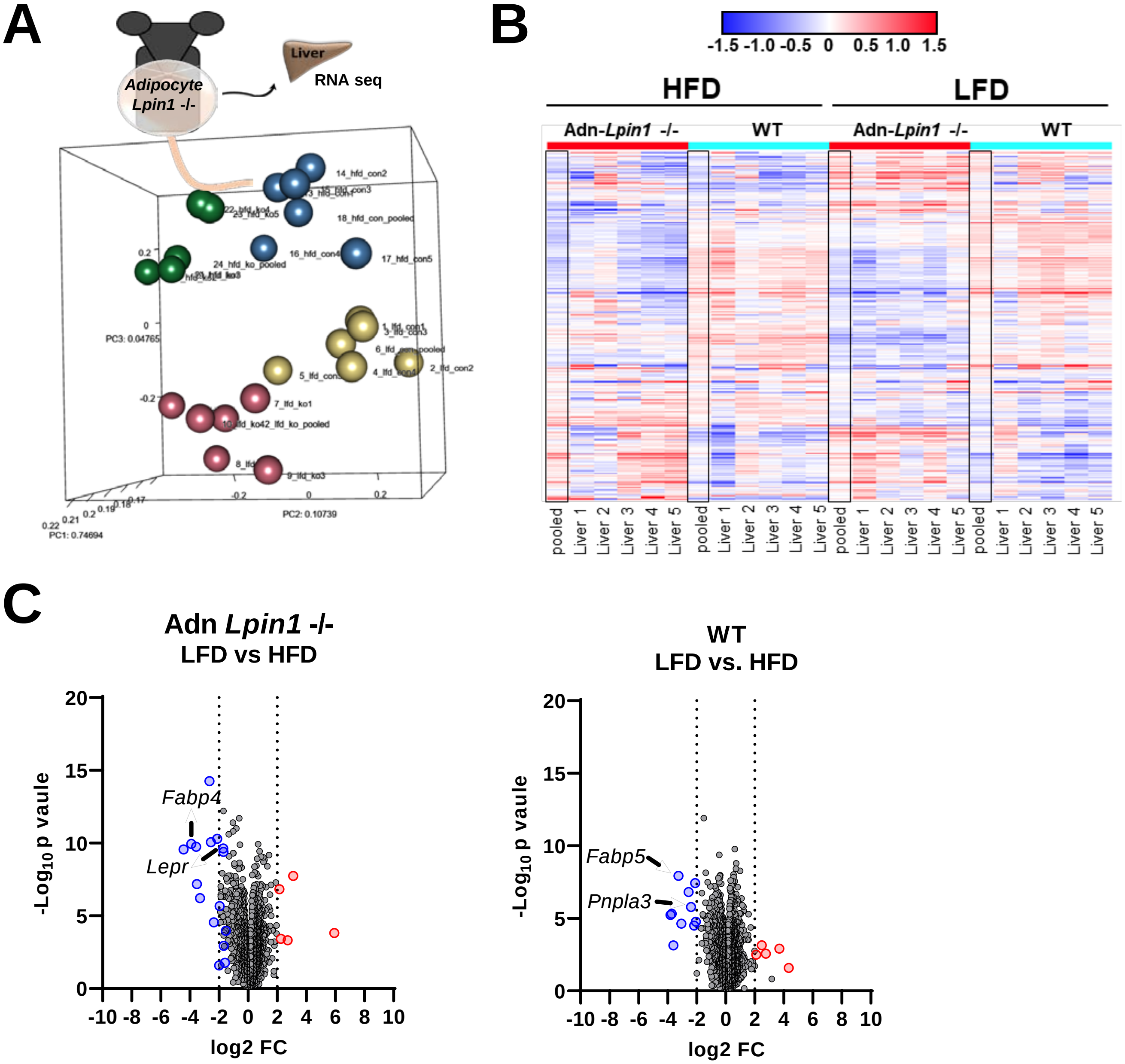

Supplemental Data Figure 5: Bulk RNA sequencing in liver.

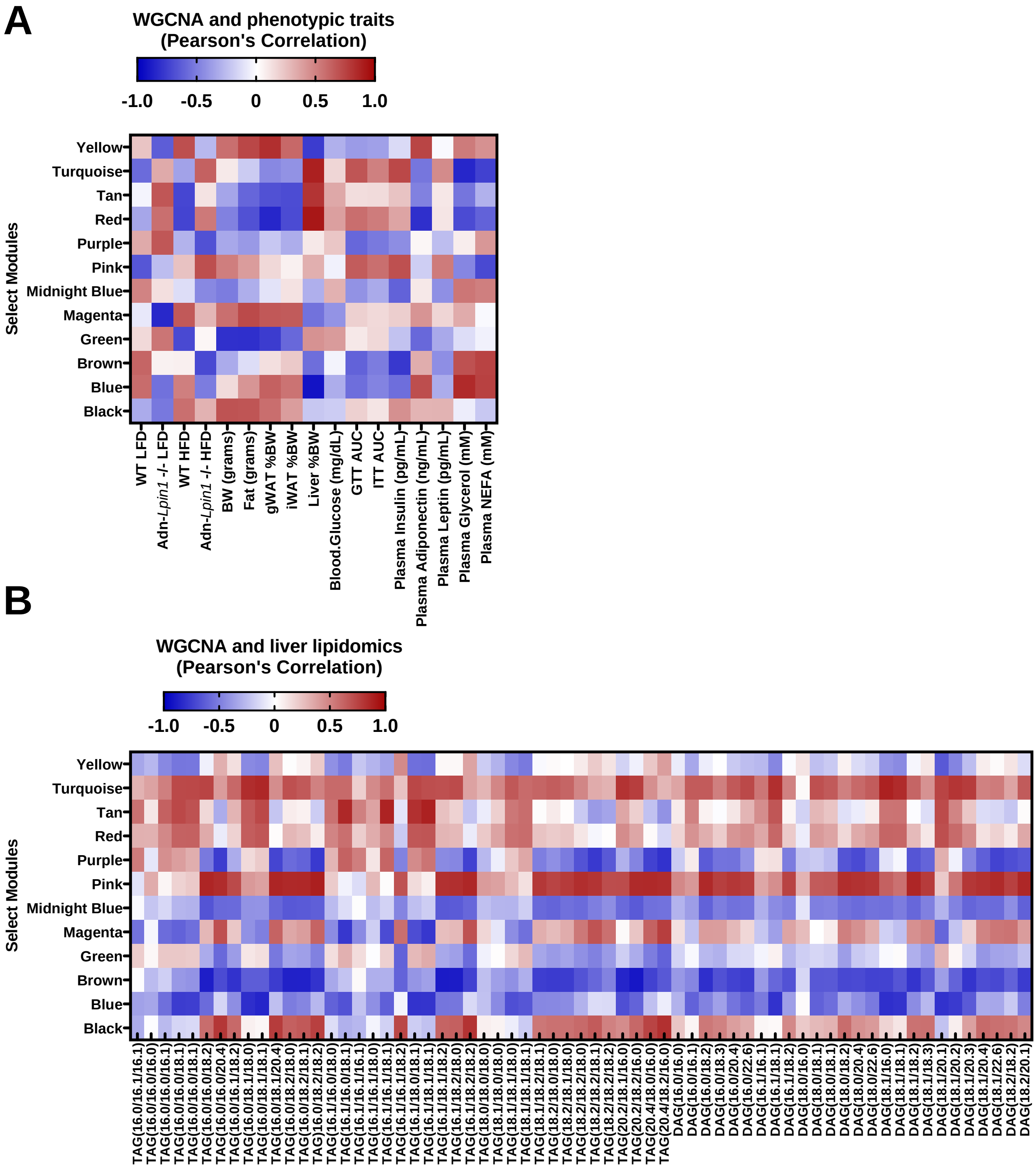
A
WGCNA and phenotypic traits
(Pearson's Correlation)
-1.0
-0.5
0
0.5
1.0
Select Modules
Yellow
Turquoise
Tan
Red
Purple
Pink
Midnight Blue
Magenta
Green
Brown
Blue
Black
WT LFD
Adn-*Lpin1* -/- LFD
WT HFD
Adn-*Lpin1* -/- HFD
BW (grams)
Fat (grams)
gWAT %BW
iWAT %BW
Liver %BW
Blood.Glucose (mg/dL)
GTT AUC
ITT AUC
Plasma Insulin (pg/mL)
Plasma Adiponectin (ng/mL)
Plasma Leptin (pg/mL)
Plasma Glycerol (mM)
Plasma NEFA (mM)
B
WGCNA and liver lipidomics
(Pearson's Correlation)
-1.0
-0.5
0
0.5
1.0
Select Modules
Yellow
Turquoise
Tan
Red
Purple
Pink
Midnight Blue
Magenta
Green
Brown
Blue
Black
TAG(16.0/16.1/16.1)
TAG(16.0/16.0/16.0)
TAG(16.0/16.0/16.1)
TAG(16.0/16.0/18.1)
TAG(16.0/16.0/18.1)
TAG(16.0/16.0/18.2)
TAG(16.0/16.0/20.4)
TAG(16.0/16.1/18.2)
TAG(16.0/18.1/18.0)
TAG(16.0/18.1/18.1)
TAG(16.0/18.1/20.4)
TAG(16.0/18.2/18.0)
TAG(16.0/18.2/18.1)
TAG)16.0/18.2/18.2)
TAG(16.1/16.0/18.0)
TAG(16.1/16.0/18.1)
TAG(16.1/16.1/16.1)
TAG(16.1/16.1/18.0)
TAG(16.1/16.1/18.1)
TAG(16.1/16.1/18.2)
TAG(16.1/18.0/18.1)
TAG(16.1/18.1/18.1)
TAG(16.1/18.1/18.2)
TAG(16.1/18.2/18.0)
TAG(16.1/18.2/18.2)
TAG(18.0/18.0/18.0)
TAG(18.1/18.0/18.0)
TAG(18.1/18.1/18.0)
TAG(18.1/18.1/18.1)
TAG(18.1/18.2/18.1)
TAG(18.2/18.0/18.0)
TAG(18.2/18.1/18.0)
TAG(18.2/18.2/18.0)
TAG(18.2/18.2/18.1)
TAG(18.2/18.2/18.2)
TAG(20.2/18.1/16.0)
TAG(20.2/18.2/16.0)
TAG(20.4/18.0/16.0)
TAG(20.4/18.2/16.0)
DAG(16.0/16.0)
DAG(16.0/16.1)
DAG(16.0/18.2)
DAG(16.0/18.3)
DAG(16.0/20.4)
DAG(16.0/22.6)
DAG(16.1/16.1)
DAG(16.1/18.1)
DAG(16.1/18.2)
DAG(18.0/16.0)
DAG(18.0/18.1)
DAG(18.0/18.1)
DAG(18.0/18.2)
DAG(18.0/20.4)
DAG(18.0/22.6)
DAG(18.1/16.0)
DAG(18.1/18.1)
DAG(18.1/18.2)
DAG(18.1/18.3)
DAG(18.1/20.1)
DAG(18.1/20.2)
DAG(18.1/20.3)
DAG(18.1/20.4)
DAG(18.1/22.6)
DAG(18.2/18.2)
DAG(18.2/20.1)

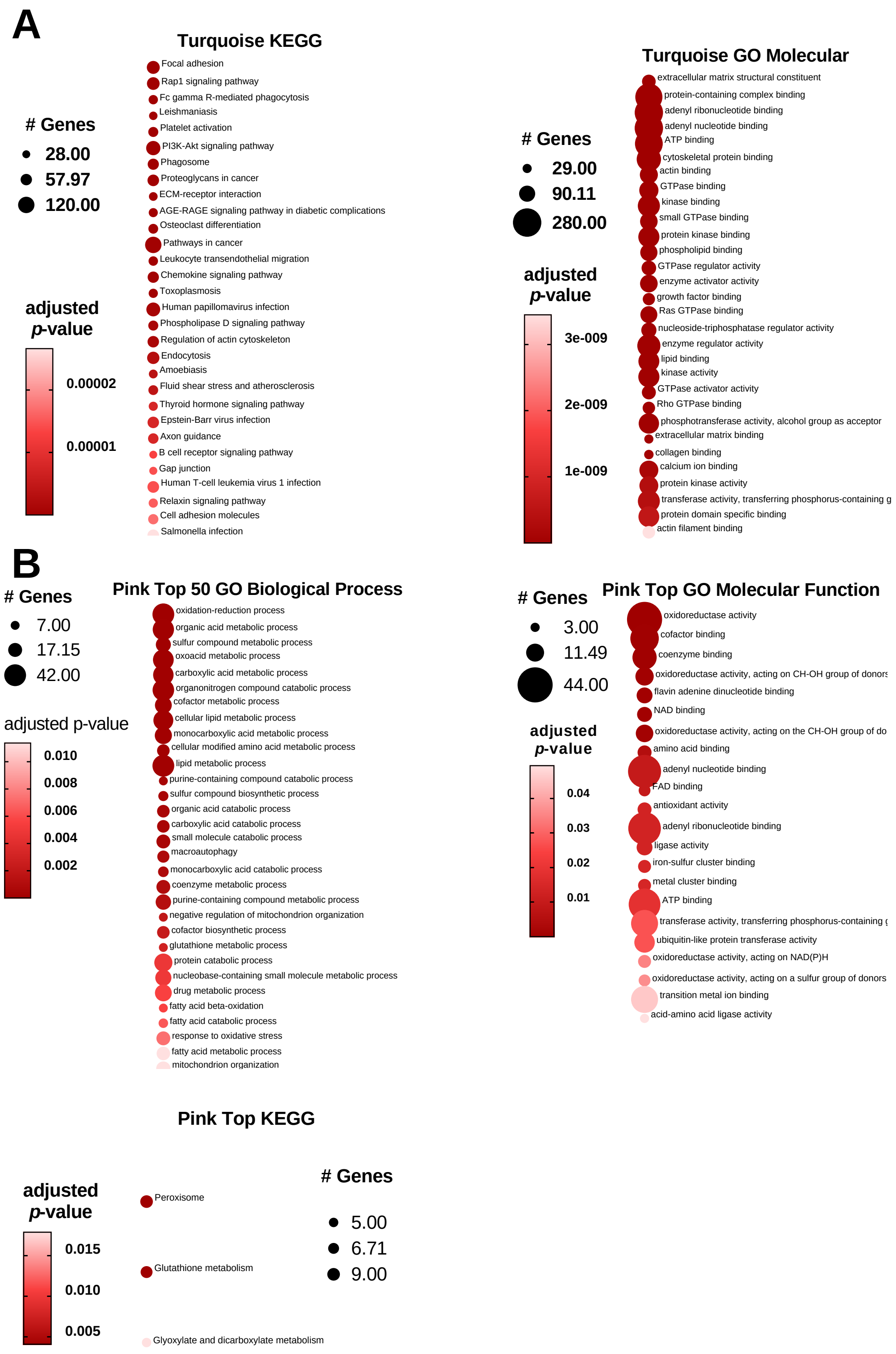

Supplemental Data Figure 7: Select WGCNA module set pathway analysis.

C

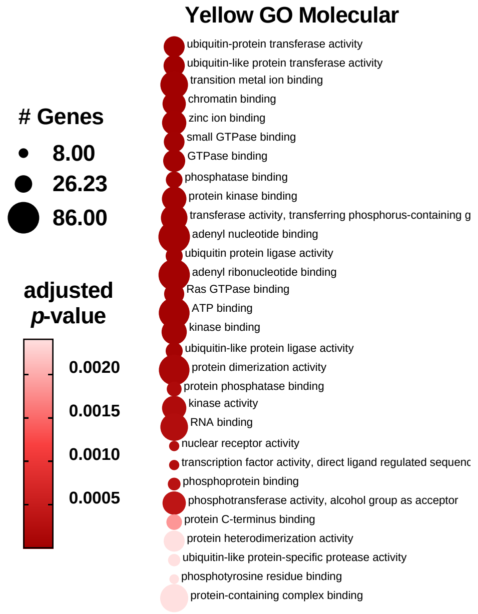

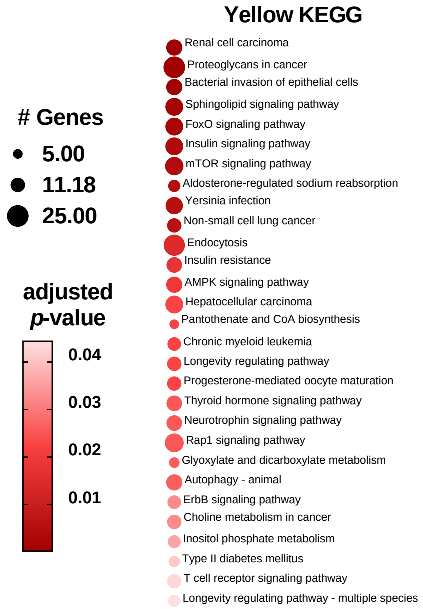

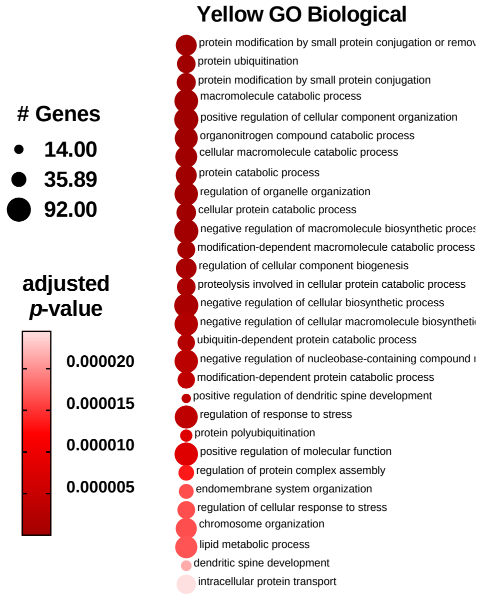

D

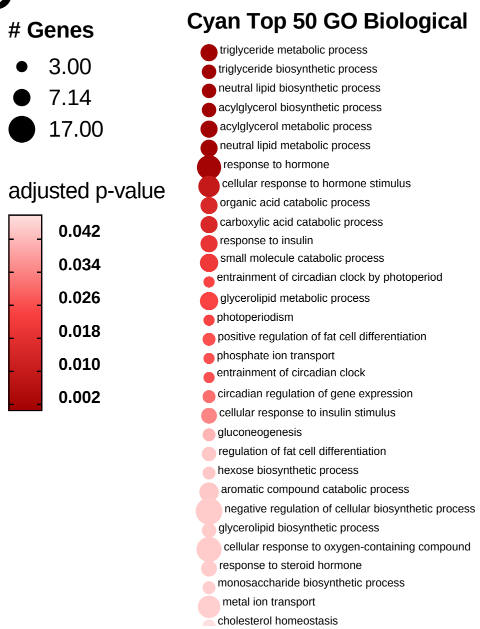

Supplemental Data Figure 7 continued: Select WGCNA module set pathway analysis.

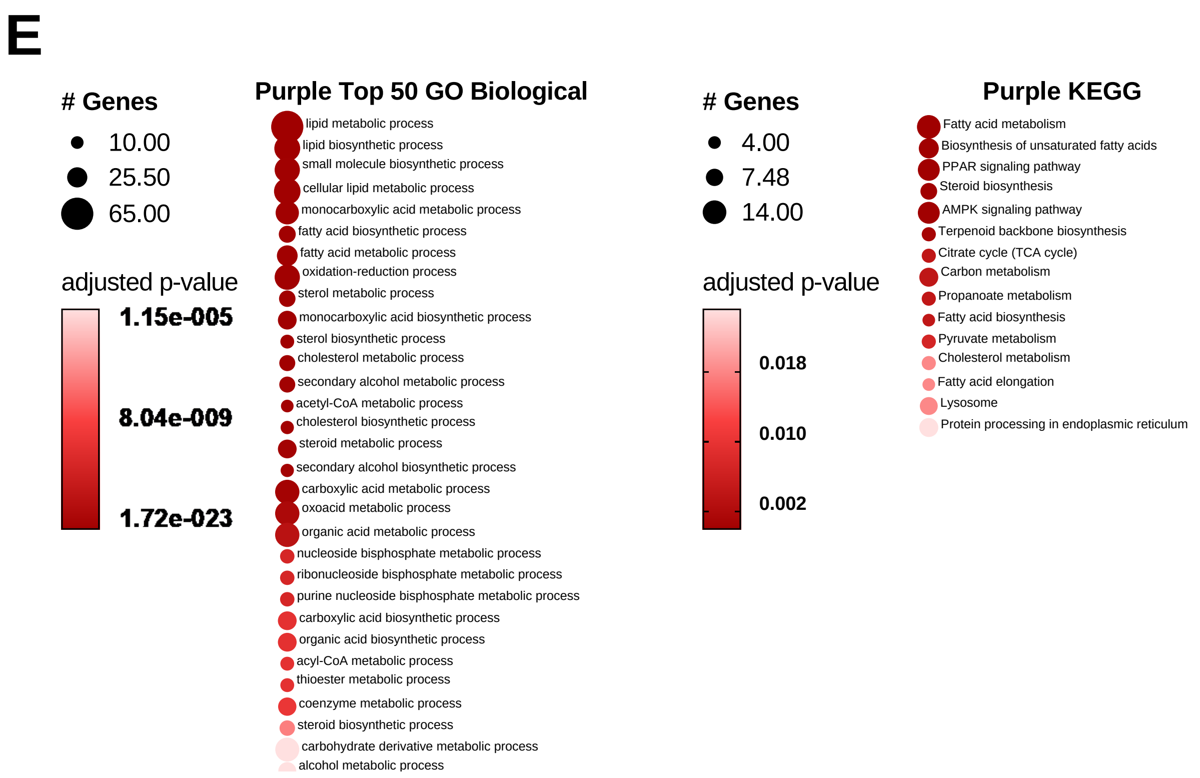

Supplemental Data Figure 7 continued: Select WGCNA module set pathway analysis.

Supplemental data Figure 8: Metabolic gene expression and their modules.

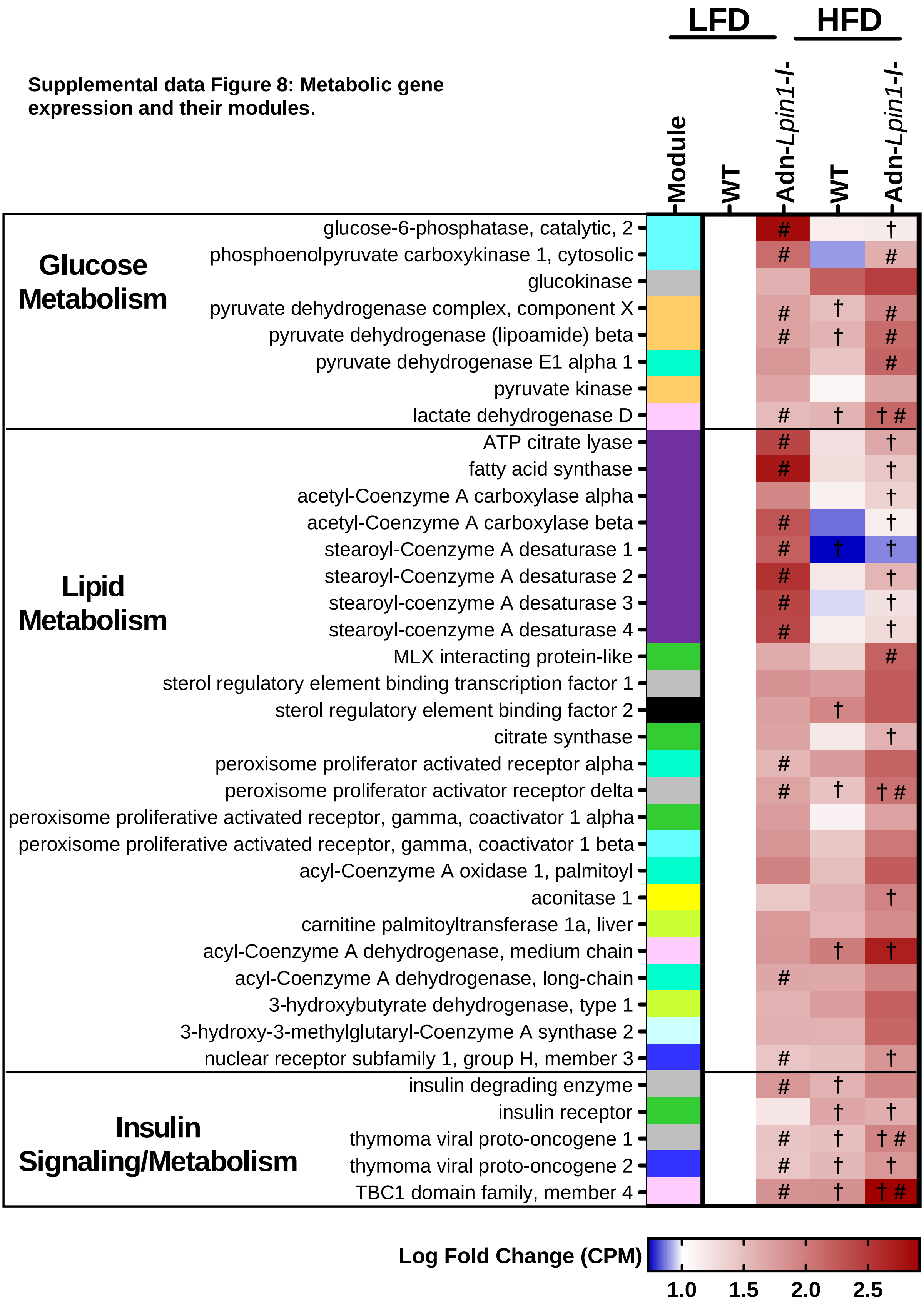

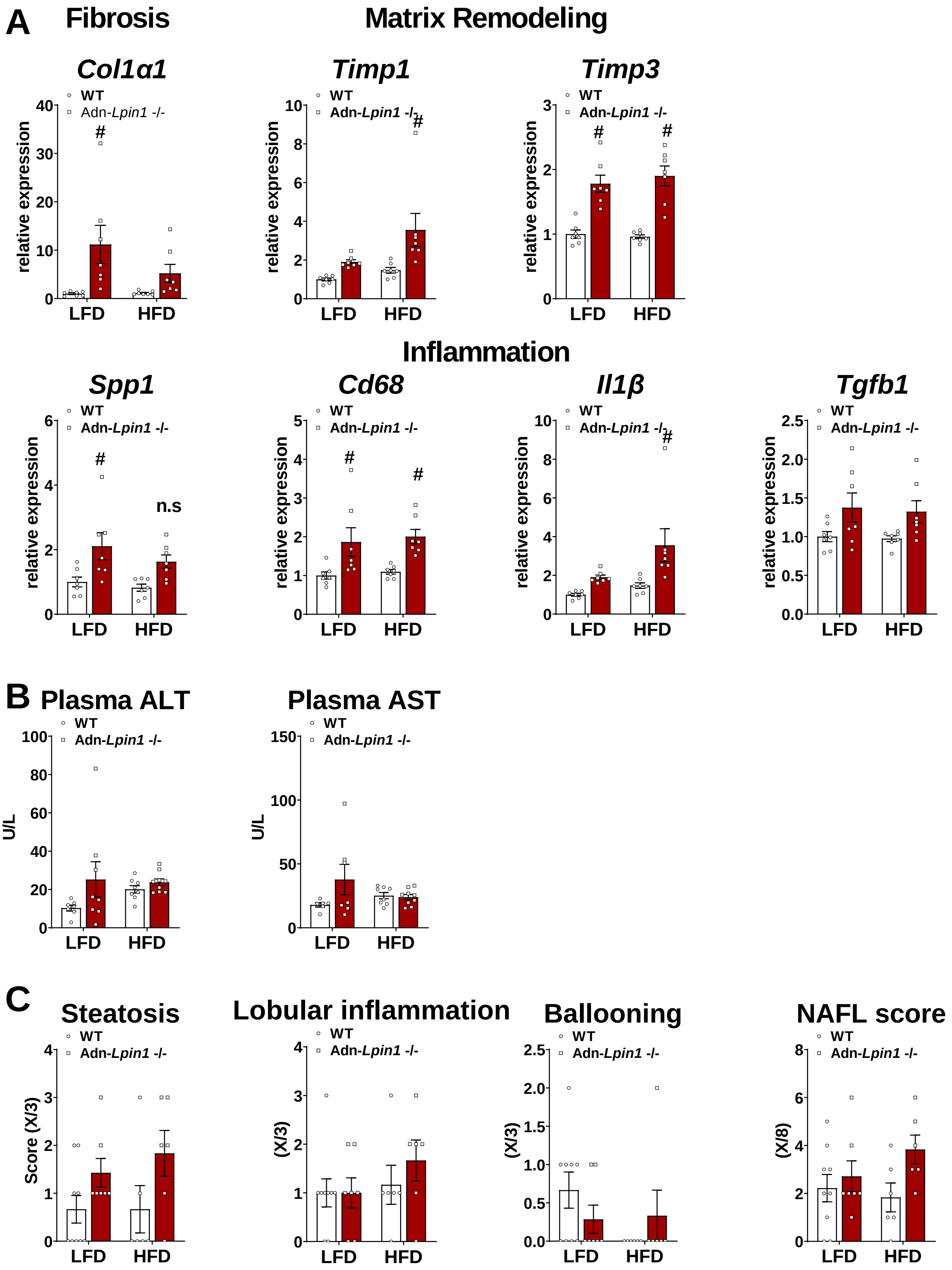

Supplemental Data Figure 9: Loss of adipocyte *Lpin1* increases early signs of hepatic fibrosis, matrix remodeling, and inflammation.

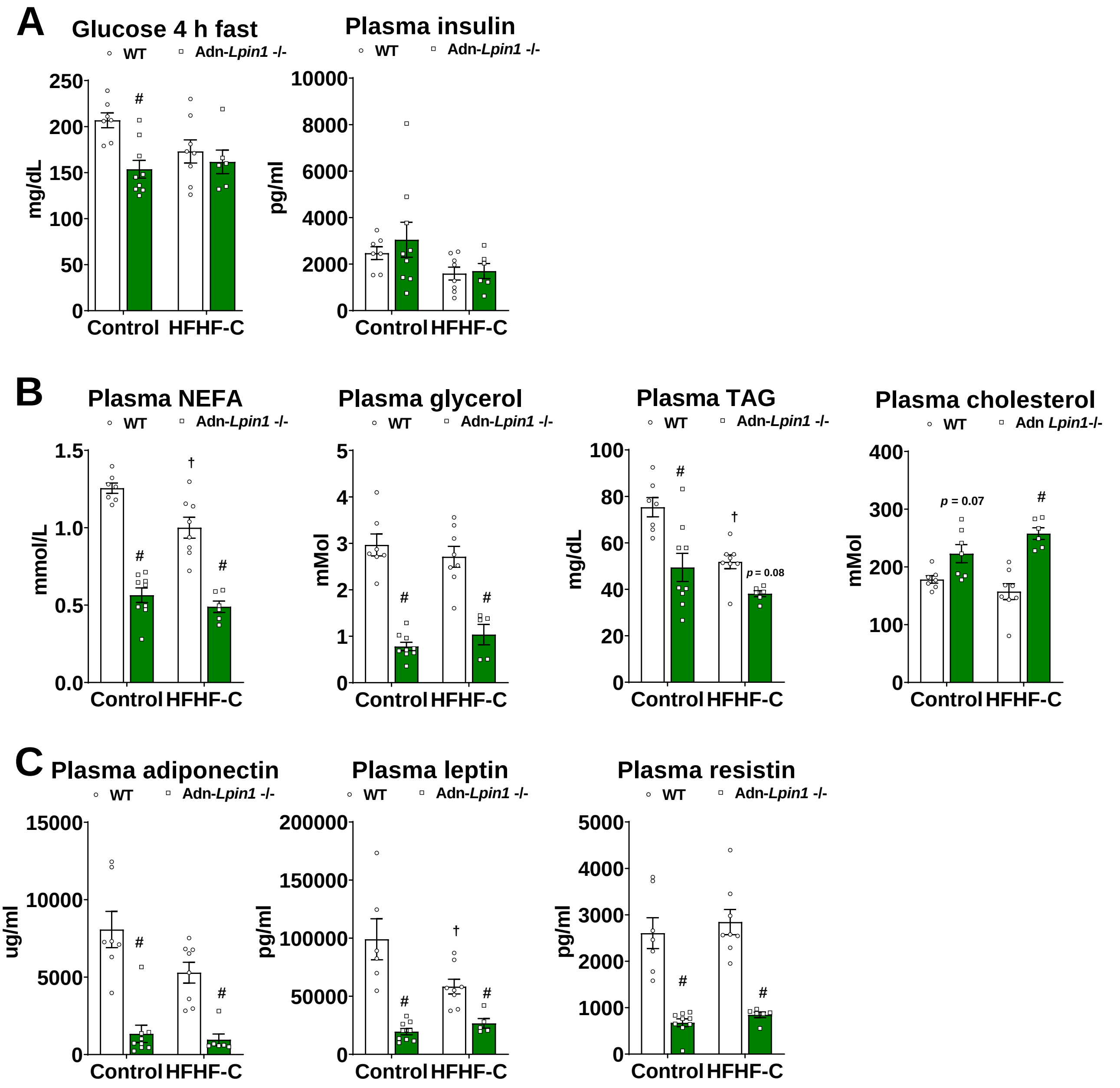

Supplemental Data Figure 10: Plasma characteristics of mice fed a NASH-inducing diet.
