# Supplementary material for: Adipocyte lipin 1 expression associates with human metabolic health and regulates systemic metabolism in mice
Source: J Clin Invest. 2024 Oct 15;134(23):e169722. doi: 10.1172/JCI169722 (PMC11601902; doi:10.1172/JCI169722)
Supplement: Unedited blot and gel images [file jci-134-169722-s066.pdf]

Complete blots for Supplemental Data Figure 1A

Lipin 1
rabbit, Santa Cruz Biotechnology, sc-98450

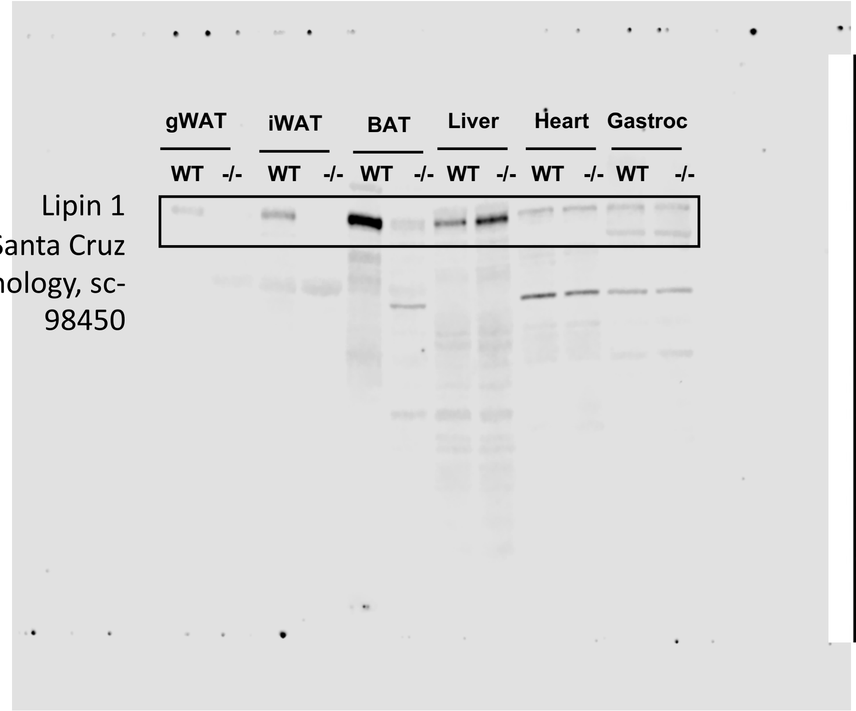

Total protein Amido Black

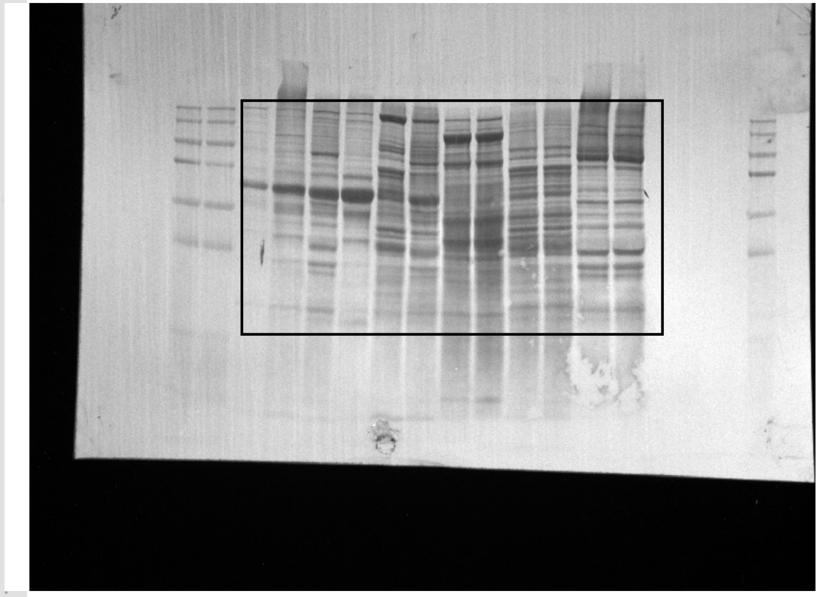

Supplemental Data Figure 1B complete blots

Lipin 1
rabbit, Santa Cruz Biotechnology, sc-98450

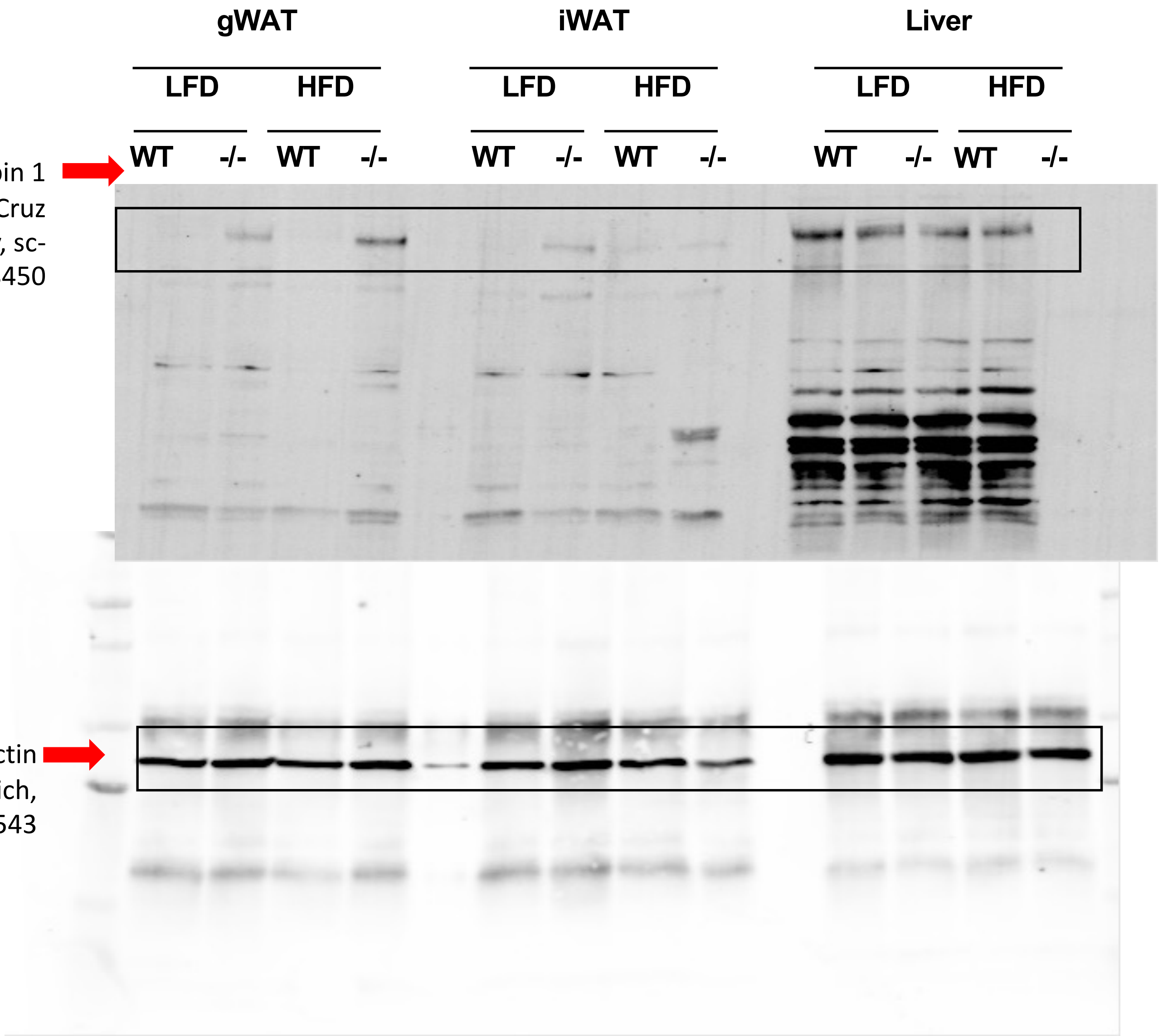

Lipin 2
rabbit, as described in PMID: 19136718

β-actin
mouse, Sigma Aldrich, SAB4502543

β-actin
mouse, Sigma Aldrich, SAB4502543

Supplemental Data Figure 1B complete blots

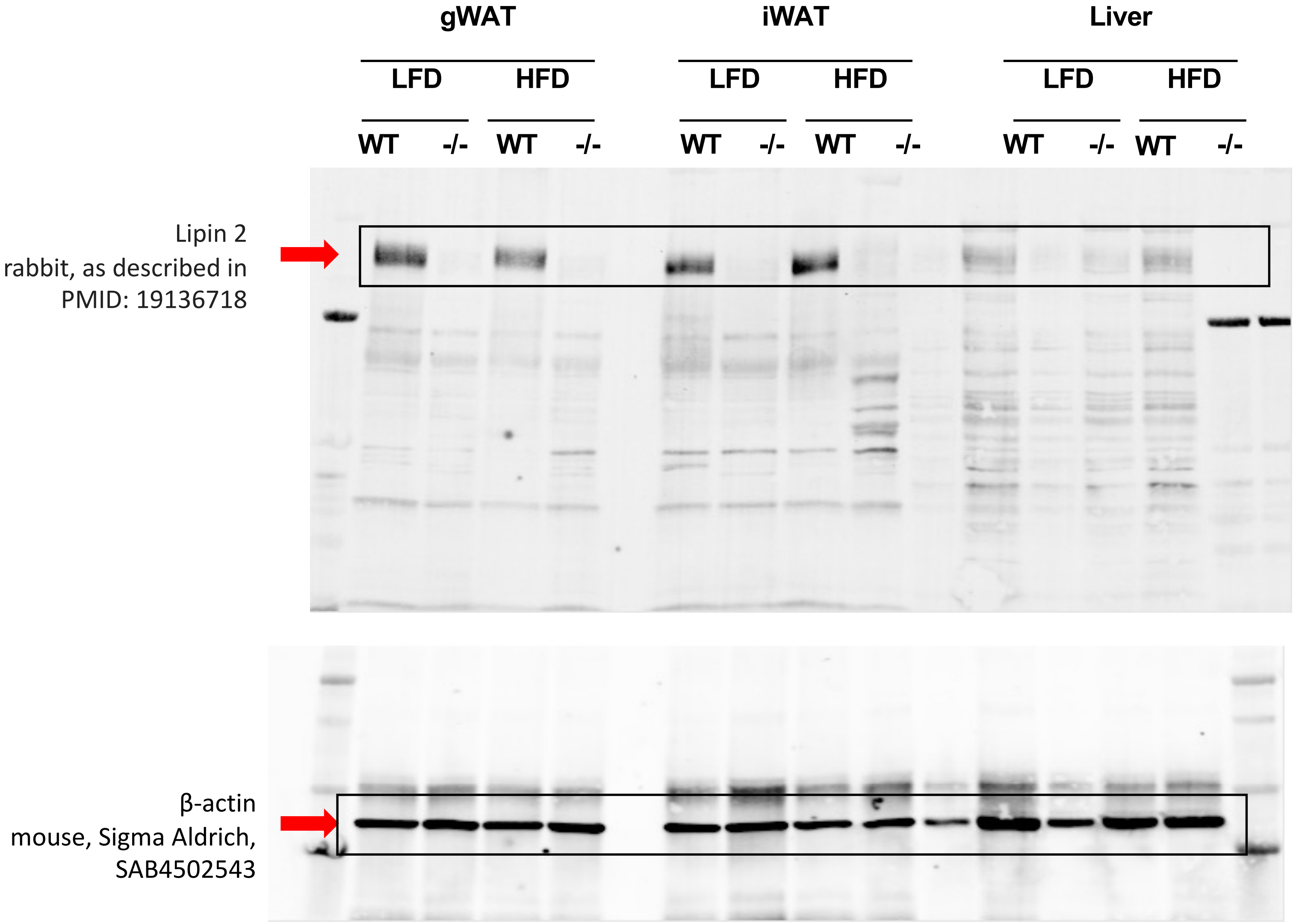

Complete blots for Supplemental Data Figure 4E

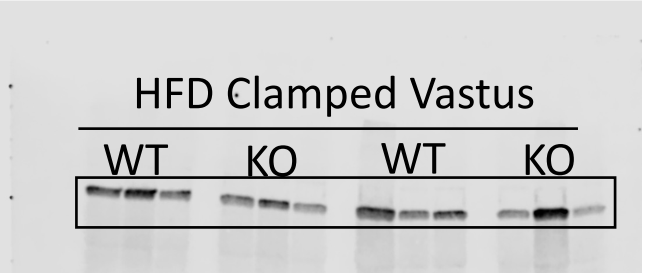

Blot 1 p-Akt, rabbit, Cell Signaling #4060

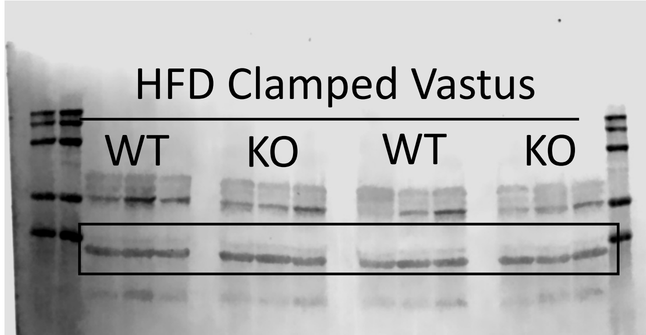

Blot 1 GAPDH, mouse, abcam ab8245

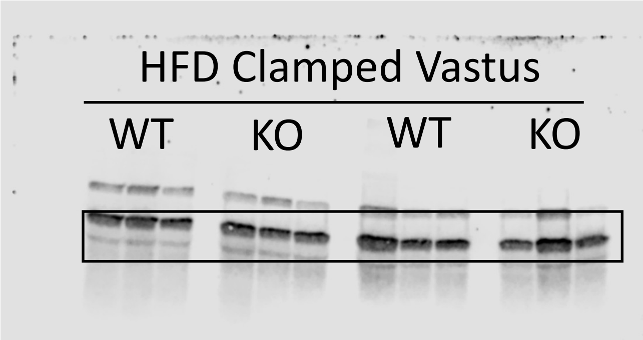

Blot 1 total GSK3B, rabbit, Cell Signaling #4337

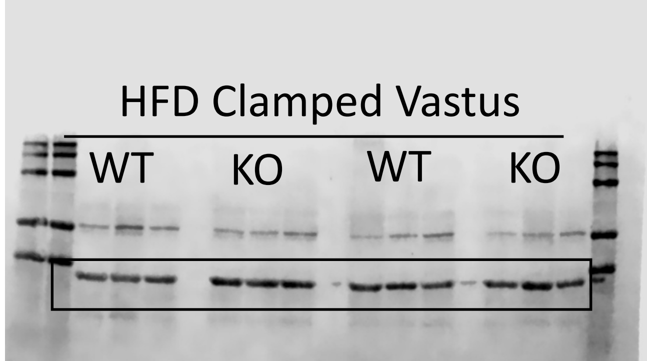

Blot 2 GAPDH, mouse, abcam ab8245

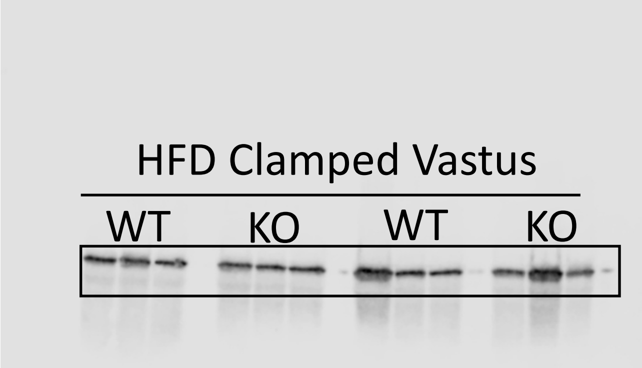

Blot 2 p-GSK3B, rabbit, Cell Signaling #5558

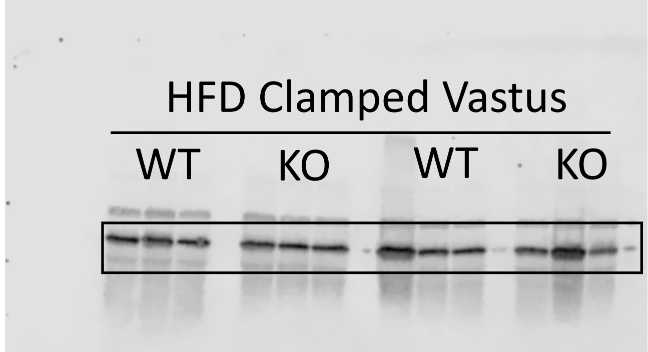

Blot 2 total Akt, rabbit, Cell Signaling #9272
